# GLP-1-directed NMDA receptor antagonism for obesity treatment

**DOI:** 10.1038/s41586-024-07419-8

**Published:** 2024-05-15

**Authors:** Jonas Petersen, Mette Q. Ludwig, Vaida Juozaityte, Pablo Ranea-Robles, Charlotte Svendsen, Eunsang Hwang, Amalie W. Kristensen, Nicole Fadahunsi, Jens Lund, Alberte W. Breum, Cecilie V. Mathiesen, Luisa Sachs, Roger Moreno-Justicia, Rebecca Rohlfs, James C. Ford, Jonathan D. Douros, Brian Finan, Bryan Portillo, Kyle Grose, Jacob E. Petersen, Mette Trauelsen, Annette Feuchtinger, Richard D. DiMarchi, Thue W. Schwartz, Atul S. Deshmukh, Morten B. Thomsen, Kristi A. Kohlmeier, Kevin W. Williams, Tune H. Pers, Bente Frølund, Kristian Strømgaard, Anders B. Klein, Christoffer Clemmensen

**Affiliations:** 1 Novo Nordisk Foundation Center for Basic Metabolic Research, Faculty of Health and Medical Sciences, University of Copenhagen, Copenhagen, Denmark; 2 Department of Drug Design and Pharmacology, Faculty of Health and Medical Sciences, University of Copenhagen, Copenhagen, Denmark; 3 Center for Hypothalamic Research, the University of Texas Southwestern Medical Center at Dallas, Dallas, TX USA; 4 Novo Nordisk Research Center Indianapolis, Indianapolis, IN USA; 5 Core Facility Pathology & Tissue Analytics, Helmholtz Munich, Neuherberg, Germany; 6 Department of Chemistry, Indiana University, Bloomington, IN USA; 7 Department of Biomedical Sciences, University of Copenhagen, Copenhagen, Denmark

**Keywords:** Obesity, Drug discovery and development

## Abstract

The *N*-methyl-d-aspartate (NMDA) receptor is a glutamate-activated cation channel that is critical to many processes in the brain. Genome-wide association studies suggest that glutamatergic neurotransmission and NMDA receptor-mediated synaptic plasticity are important for body weight homeostasis^1^. Here we report the engineering and preclinical development of a bimodal molecule that integrates NMDA receptor antagonism with glucagon-like peptide-1 (GLP-1) receptor agonism to effectively reverse obesity, hyperglycaemia and dyslipidaemia in rodent models of metabolic disease. GLP-1-directed delivery of the NMDA receptor antagonist MK-801 affects neuroplasticity in the hypothalamus and brainstem. Importantly, targeting of MK-801 to GLP-1 receptor-expressing brain regions circumvents adverse physiological and behavioural effects associated with MK-801 monotherapy. In summary, our approach demonstrates the feasibility of using peptide-mediated targeting to achieve cell-specific ionotropic receptor modulation and highlights the therapeutic potential of unimolecular mixed GLP-1 receptor agonism and NMDA receptor antagonism for safe and effective obesity treatment.

## Abstract (web summary)

Unimolecular integration of NMDA receptor antagonism with GLP-1 receptor agonism effectively reverses obesity, hyperglycaemia and dyslipidaemia in rodent models of metabolic disease.

## Main

Non-competitive, open-channel NMDA receptor blockers are used clinically for the management of Alzheimer’s disease and treatment-resistant depression^2,3^. It is believed that this class of small-molecule drugs improves brain disorders through mechanisms involving neurostructural changes and synaptic plasticity^4^. Notably, genome-wide association study (GWAS) analyses for body mass index (BMI) have linked glutamatergic signalling and NMDA receptor-related neuroplasticity to regulation of body weight and obesity^1,5^. In rodents, disparate effects of NMDA receptor antagonism on food intake have been observed. Specifically, antagonizing NMDA receptors in the brainstem has been associated with an increase in short-term food intake^6,7^; by contrast, antagonizing NMDA receptors in the hypothalamus has been linked to a reduction in food intake and a decrease in body weight^8^. In the context of prolonged systemic administration, NMDA receptor antagonists, such as memantine and MK-801 (also known as dizocilpine), induce anorexia and weight loss in rodents^9–11^. Furthermore, these antagonists are reported to diminish palatable food preferences in rodents^12^ and non-human primates^13^ and to reduce binge-eating episodes in humans^14^.

**Extended Data Fig. 1:**
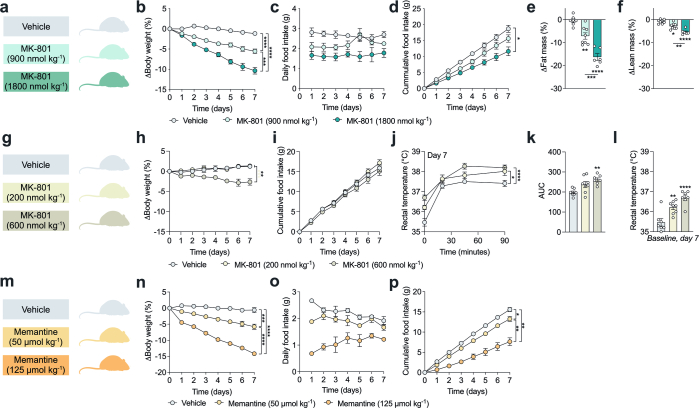
Pharmacological NMDA receptor antagonism dose-dependently lowers body weight but induces hyperthermia. **a**-**f**, Treatment of C57BL/6 J DIO mice with once-daily subcutaneous (s.c.) injections of 900 nmol kg^−1^ MK-801 (n = 8 mice and n = 4 cages), 1800 nmol kg^−1^ MK-801 (n = 6 mice and n = 3 cages) or vehicle (isotonic saline, n = 7 mice and n = 3 cages) over 7 days. One mouse and cage were excluded from vehicle group due to the development of constipation. **a**, Schematic. **b**, Change in body weight. **c**, Daily food intake. **d**, Cumulative food intake. **e**, Change in fat mass. **f**, Change in lean mass. **g**-**l**, Treatment of C57BL/6 J DIO mice with once-daily s.c. injections of 200 nmol kg^−1^ MK-801 (n = 8 mice and n = 4 cages), 600 nmol kg^−1^ MK-801 (n = 7 mice and n = 4 cages) or vehicle (isotonic saline, n = 7 mice and n = 4 cages) for 7 days. **g**, Schematic. **h**, Change in body weight. **i**, Cumulative food intake. **j**, Change in rectal temperature in response to treatment on day 7. **k**, Area under curve (AUC) of **j**. **l**, Baseline rectal temperature on day 7. **m**-**p**, Treatment of C57BL/6 J DIO mice with once-daily s.c. injections of 50 µmol kg^−1^ memantine (n = 6 mice and n = 3 cages), 125 µmol kg^−1^ memantine (n = 6 mice and n = 3 cages) or vehicle (isotonic saline, n = 6 mice and n = 3 cages) over 7 days. **m**, Schematic. **n**, Change in body weight. **o**, Daily food intake. **p**, Cumulative food intake. Data analysed by one-way ANOVA, multiple comparison, Bonferroni post hoc test (**e**, **f**, **k** and **l**) and two-way repeated measures ANOVA to assess main effects of treatment (**b**, **d**, **h**-**j**, **n** and **p**). Data represents mean ± SEM; ^*^P < 0.05, ^**^P < 0.01, ^***^P < 0.001, ^****^P < 0.0001. Detailed statistics are in Supplementary Table 1. The diagrams in **a**, **g** and **m** were created using BioRender. Source Data

Here we affirm the pharmacological potential of NMDA receptor channel blockers for obesity treatment (Extended Data Fig. 1a–p). Once-daily subcutaneous (s.c.) injections of the potent NMDA receptor antagonist MK-801 in diet-induced obese (DIO) mice led to a dose-dependent decrease in food intake and body weight (Extended Data Fig. 1a–f). MK-801 is widely used experimentally, but its clinical application is hampered by severe adverse effects. For example, chronic treatment with MK-801 promotes hyperthermia and hyperlocomotion^15–17^. Consistent with this, we demonstrate that treatment with MK-801, even at doses that do not evoke weight loss, elicits pronounced hyperthermia in DIO mice (Extended Data Fig. 1g–l), emphasizing the unsuitability of MK-801 monotherapy for obesity treatment.

## Development of GLP-1–MK-801

**Extended Data Fig. 2:**
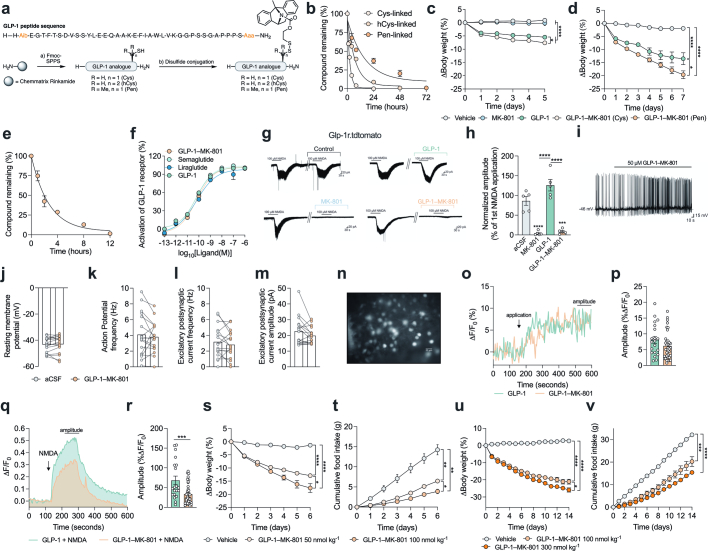
Pharmacological characterization of GLP-1–MK-801. **a**, Chemical synthesis of GLP-1–MK-801 conjugates with different cysteine homologues. **b**, In vitro stability assay in human plasma. The assay was performed as biological replicates of GLP-1–MK-801 (Cys-linked) (*n* = 3), GLP-1–MK-801 (hCys-linked) (*n* = 2), GLP-1–MK-801 (Pen-linked) (*n* = 3). **c**, Change in body weight of DIO mice treated with once-daily s.c. injections of 100 nmol kg^−1^ MK-801 (*n* = 8 mice), 100 nmol kg^−1^ GLP-1 (*n* = 8 mice), 100 nmol kg^−1^ GLP-1–MK-801 (Cys-linked) (*n* = 8 mice) or vehicle (isotonic saline, *n* = 8 mice) for 5 days. **d**, Change in body weight of DIO mice treated with once-daily s.c. injections of 100 nmol kg^−1^ GLP-1 (*n* = 6 mice), 100 nmol kg^−1^ GLP-1–MK-801 (Pen-linked) (*n* = 6 mice) or vehicle (isotonic saline, *n* = 6 mice) for 7 days. **e**, Degradation assay. GLP-1–MK-801 was incubated in PBS with 200 mM glutathione at pH 7.0. The assay was performed as triplicates (*n* = 3). **f**, Murine GLP-1 receptor activation of GLP-1, GLP-1–MK-801, liraglutide and semaglutide in transiently transfected HEK293 cells. The data represents dose-response at 2 min and is normalized to the maximal GLP-1 response (100%). The assay was performed as duplicates (*n* = 2). **g**, **h**, Electrophysiological recordings of GLP-1 receptor-positive neurons, which were identified using a tdtomato reporter, being stimulated with NMDA after 30 min of bath application of GLP-1 or GLP-1–MK-801. **g**, Current responses at a holding potential of −70 mV elicited by NMDA bath application in GLP-1 receptor-positive neurons of the arcuate nucleus (ARC) with bath application of 50 µM GLP-1–MK-801, 50 µM GLP-1, 50 µM MK-801 or artificial cerebrospinal fluid (aCSF). **h**, Bar graph summarizing the effect of NMDA-induced inward current after control (aCSF, *n* = 5 neurons), GLP-1 (50 μM, *n* = 5 neurons), MK-801 (50 μM, *n* = 5 neurons) or GLP-1–MK-801 (50 μM, *n* = 5 neurons). Bath application normalized to the first bath application of NMDA (100 μM). **i**-**m**, Electrophysiological recordings of POMC neurons stimulated with GLP-1–MK-801. **i**, Representative trace showing that 50 μM GLP-1–MK-801 acute bath application induces a depolarization of POMC neurons in ARC (*n* = 6 of 17 neurons). Bar graphs summarizing the acute effect of 50 μM GLP-1–MK-801 on POMC neurons. **j**, Resting membrane potential, **k**, Action potential frequency *n*. **l**, Excitatory postsynaptic current (EPSC) frequency. **m**, EPSC amplitude of POMC neurons that were depolarized in response to GLP-1–MK-801 bath application. **n**-**r**, Calcium imaging of ARC brain slices. **n**, Representative image of Fura-2AM loaded cells in an ARC brain slice. Scalebar is 20 µm. **o**, Application of GLP-1 and GLP-1–MK-801 induced changes in fluorescence indicative of rises in intracellular calcium (%ΔF/F_0_) as shown by these representative fluorescent responses of two different Fura 2-AM loaded cells to application of GLP-1 (1 µM) or GLP-1–MK-801 (1 µM). **p**, The amplitude of the change in fluorescence (%ΔF/F_0_) elicited by GLP-1 (1 µM, *n* = 20 neurons) or GLP-1–MK-801 (1 µM, *n* = 38 neurons). **q**, Representative traces of intracellular calcium levels in response to application of NMDA (50 µM) following bath application of GLP-1 (1 µM) or GLP-1–MK-801 (1 µM). **r**, Quantification of NMDA-induced (50 µM) intracellular calcium rises (%ΔF/F_0_) following bath application of GLP-1 (1 µM, *n* = 20 neurons) or GLP-1–MK-801 (1 µM, *n* = 38 neurons). **s**, **t**, Treatment of DIO mice with once-daily s.c. injections of 50 nmol kg^−1^ GLP-1–MK-801 (*n* = 6 mice and *n* = 3 cages), 100 nmol kg^−1^ GLP-1–MK-801 (*n* = 6 mice and *n* = 3 cages) or vehicle (isotonic saline, *n* = 6 mice and *n* = 3 cages) for 6 days. **s**, Change in body weight. **t**, Cumulative food intake. **u**, **v**, Treatment of C57BL/6 J DIO mice with once-daily s.c. injections of 100 nmol kg^−1^ GLP-1–MK-801 (*n* = 8 mice and *n* = 4 cages), 300 nmol kg^−1^ GLP-1–MK-801 (*n* = 8 mice and *n* = 4 cages) or vehicle (isotonic saline, *n* = 8 mice and *n* = 4 cages) for 14 days. **u**, Change in body weight. **v**, Cumulative food intake. Data analysed by paired two-tailed Student’s t-test (**j**-**m**), unpaired two-tailed Student’s t-test (**p** and **r**), one-way ANOVA, multiple comparison, Bonferroni *post hoc* test (**h**) and two-way repeated measures ANOVA to assess main effects of treatment (**c**, **d** and **s**-**v**). Data represents mean ± SEM; ^*^P < 0.05, ^**^P < 0.01, ^***^P < 0.001, ^****^P < 0.0001. Detailed statistics are in Supplementary Table 1. Source Data

To safely harness the weight-lowering properties of NMDA receptor antagonism, we developed a targeting approach based on the hypothesis that conjugation of MK-801 to a GLP-1 analogue through a chemically cleavable disulfide linker would enhance the therapeutic potential of NMDA receptor antagonism in appetite-regulating brain regions enriched for GLP-1 receptors, while mitigating the adverse effects related to the non-restricted actions of MK-801. These efforts involve chemically masking the secondary amine of MK-801 to render it inactive until the linker is cleaved, by the higher concentration of thiol-containing compounds in the intracellular compartment, resulting in liberation of MK-801^18^. Through iterative chemical synthesis and structural refinement, we developed a stabilized conjugate equipped with a C-terminal l-penicillamine residue and a self-immolative disulfide linker (GLP-1–MK-801) (Extended Data Fig. 2a). The plasma stability of GLP-1–MK-801 was optimized through incorporation of different cysteine homologues to the C terminus of the peptide to facilitate disulfide conjugation to the linker (Extended Data Fig. 2b–d). Moreover, we confirmed that the conjugate is degraded in vitro by incubation under high concentrations of glutathione (Extended Data Fig. 2e). We observed that GLP-1–MK-801 has similar receptor signalling properties at the GLP-1 receptor compared to the parent GLP-1 analogue as well as to the pharmacokinetically optimized GLP-1 receptor agonists semaglutide and liraglutide (Extended Data Fig. 2f). Furthermore, we confirmed GLP-1–MK-801-mediated target engagement with NMDA receptors in GLP-1-receptor-positive neurons in the arcuate nucleus using electrophysiological recordings of isolated NMDA receptor currents. Specifically, we demonstrated that GLP-1–MK-801, but not the parent GLP-1 analogue, suppressed NMDA-induced inward currents (Extended Data Fig. 2g,h). In agreement with previous research on GLP-1 receptor agonists, GLP-1–MK-801 increased the excitability of around 35% of POMC-expressing neurons^19^ (POMC neurons; Extended Data Fig. 2i–m). These observations were corroborated by single-cell calcium imaging studies in slices of arcuate nuclei, demonstrating that GLP-1–MK-801 inhibits the NMDA-induced intracellular calcium surge relative to GLP-1 alone (Extended Data Fig. 2n–r).

## Metabolic phenotyping of GLP-1–MK-801

**Fig. 1:**
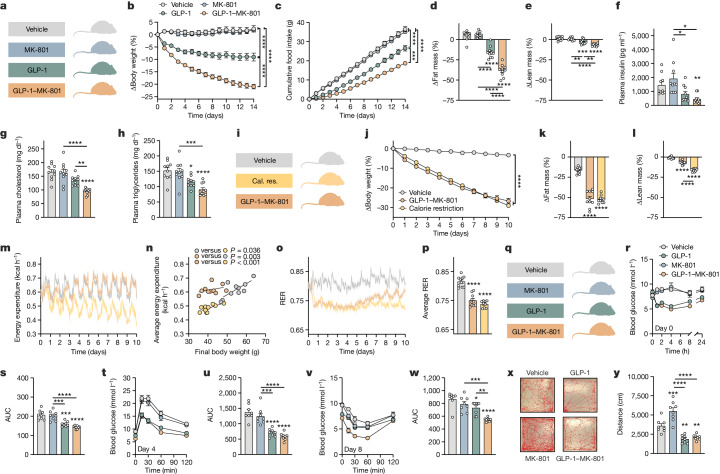
GLP-1–MK-801 corrects metabolic disease. **a**–**h**, DIO mice were treated once-daily with s.c. injections of MK-801, GLP-1, GLP-1–MK-801 or vehicle for 14 days. *n* = 10 mice per group. 100 nmol kg^−1^ dose. **a**, Schematic. **b**, Change in body weight. **c**, Cumulative food intake. **d**, Change in fat mass. **e**, Change in lean mass. **f**, Plasma insulin. **g**, Plasma cholesterol. **h**, Plasma triglycerides. **i**–**p**, DIO mice were treated once-daily with s.c. injections of GLP-1–MK-801 (100 nmol kg^−1^), calorie restriction (cal. res.) to match the weight loss of the GLP-1–MK-801 group or vehicle for 10 days. *n* = 9–10 mice per group. **i**, Schematic. **j**, Change in body weight. **k**, Change in fat mass. **l**, Change in lean mass. **m**, Energy expenditure. **n**, Average energy expenditure relative to final body weight. **o**, RER. One mouse in the calorie-restriction group was excluded due to a CO_2_-sensor-related deviation. **p**, Average RER. **q**–**w**, DIO mice were treated once-daily with s.c. injections of MK-801, GLP-1, GLP-1–MK-801 or vehicle for 8 days. *n* = 8 mice per group. 100 nmol kg^−1^ dose. **q**, Schematic. **r**, Compound tolerance test on day 0. **s**, Area under the curve (AUC) of data in **r**. **t**, Glucose tolerance test on day 4. **u**, AUC of data in **t**. **v**, Insulin tolerance test on day 8. **w**, AUC of data in **v**. **x**,**y**, Open-field test after a single s.c. injection of MK-801, GLP-1, GLP-1–MK-801 or vehicle. *n* = 8 mice per group. 300 nmol kg^−1^ dose. **x**, Representative traces. **y**, Distance travelled. Data were analysed using one-way analysis of variance (ANOVA) with Bonferroni post hoc multiple-comparison test (**d**–**h**, **k**, **l**, **p**, **s**, **u**, **w** and **y**), two-way repeated-measures ANOVA to assess main effects of treatment (**b**, **c** and **j**) or analysis of covariance (ANCOVA) computed with calR using body weight as a covariate (**n**). Data are mean ± s.e.m. *P < 0.05, **P < 0.01, ***P < 0.001, ****P < 0.0001. Detailed statistics are provided in Supplementary Table 1. The diagrams in **a**, **i** and **q** were created using BioRender. Source Data

**Extended Data Fig. 3:**
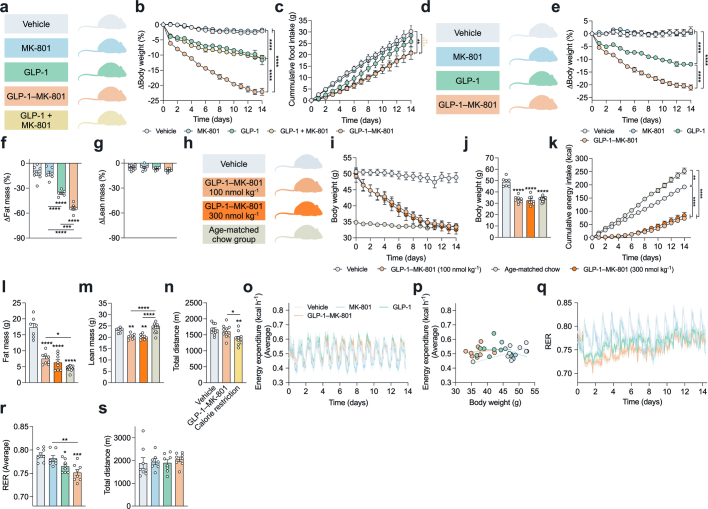
Supplementary metabolic phenotyping of GLP-1–MK-801. **a**-**c**, Treatment of DIO mice with once-daily s.c. injections of 100 nmol kg^−1^ MK-801 (*n* = 7 mice and *n* = 4 cages), 100 nmol kg^−1^ GLP-1 (*n* = 6 mice and *n* = 3 cages), 100 nmol kg^−1^, GLP-1–MK-801 (*n* = 6 mice and *n* = 3 cages), co-administration of 100 nmol kg^−1^ GLP-1 and 100 nmol kg^−1^ MK-801 (*n* = 6 mice and *n* = 3 cages) or vehicle (isotonic saline, *n* = 7 mice and *n* = 4 cages) for 14 days. **a**, Schematic. **b**, Change in body weight. **c**, Cumulative food intake. **d**-**g** and **o**-**s**, Treatment of DIO mice in metabolic cages with once-daily s.c. injections of 100 nmol kg^−1^ MK-801 (*n* = 7-8 mice), 100 nmol kg^−1^ GLP-1 (*n* = 8 mice), 100 nmol kg^−1^ GLP-1–MK-801 (*n* = 8 mice) or vehicle (isotonic saline, *n* = 8 mice) for 14 days. **d**, Schematic. **e**, Change in body weight. **f**, Change in fat mass. For MRI, two values were not registered from MK-801 group, two values were not registered from GLP-1 group and two values were not registered from GLP-1–MK-801 group due to MRI instrumental error. **g**, Change in lean mass. **h**-**m**, GLP-1–MK-801 corrects body weight and fat mass relative to age-matched chow-fed control mice. Treatment of DIO mice with once-daily s.c. injections of 100 nmol kg^−1^ GLP-1–MK-801 (*n* = 8 mice and *n* = 4 cages), 300 nmol kg^−1^ GLP-1–MK-801 (*n* = 8 mice and *n* = 4 cages) or vehicle (isotonic saline, *n* = 7 mice and *n* = 4 cages) in comparison with vehicle-treated age-matched chow-fed C57BL/6 J mice (*n* = 16 mice and *n* = 8-11 cages) for 14 days. Three double-housed cages with age-matched control animals were split on day 7 of the experiment due to fighting. One mouse was found dead in vehicle group. **h**, Schematic. **i**, Change in body weight. **j**, Body weight on day 14. **k**, Cumulative food intake. **l**, Fat mass on day 14. **m**, Lean mass on day 14. **n**, Total locomotor activity of mice from indirect calorimetry study in Fig. 1i–p. **o**, Energy expenditure. One mouse in MK-801 group excluded due to sensor related deviation. **p**, Regression-based analysis of energy expenditure relative to body weight on day 14. **q**, Respiratory exchange ratio (RER). **r**, Average respiratory exchange ratio. **s**, Total locomotor activity. Data analysed by one-way ANOVA, multiple comparison, Bonferroni *post hoc* test (**f**, **g**, **j**, **l**-**n**, **r** and **s**), two-way repeated measures ANOVA to assess main effects of treatment (**b**, **c**, **e** and **k**), and ANCOVA computed with calR using body weight as a covariate (**p**). Data represents mean ± SEM; ^*^P < 0.05, ^**^P < 0.01, ^***^P < 0.001, ^****^P < 0.0001. Detailed statistics are in Supplementary Table 1. The diagrams in **a**, **d** and **h** were created using BioRender. Source Data

After the dose-determination studies (Extended Data Fig. 2s–v), we assessed the in vivo metabolic effects in DIO mice by comparing GLP-1–MK-801 with the parent GLP-1 analogue, MK-801 and vehicle treatments (Fig. 1a). Over a 14-day treatment period, GLP-1–MK-801 synergistically lowered body weight compared with the dose-matched monotherapies and produced a vehicle-corrected weight loss of 23.2% (Fig. 1b and Extended Data Fig. 3a,b,d,e). The potent weight loss induced by GLP-1–MK-801 was linked to a potentiated decrease in food intake in mice treated with GLP-1–MK-801 compared to mice treated with vehicle or GLP-1 or MK-801 monotherapies (Fig. 1c and Extended Data Fig. 3c). GLP-1–MK-801 produced a vehicle-corrected reduction in body fat mass of 45%, accompanied by an 8% loss in lean mass (Fig. 1d,e and Extended Data Fig. 3f,g). In comparison, GLP-1 induced a 22% decrease in body fat mass and a 4% reduction in lean mass. Mice treated with GLP-1–MK-801 for 14 days exhibited lower plasma insulin levels compared with both vehicle-treated mice and those treated with MK-801 alone (Fig. 1f). Moreover, mice receiving GLP-1 monotherapy displayed significantly lower insulin levels compared with mice that were treated with MK-801 alone. Treatment with GLP-1–MK-801 led to a decrease in plasma cholesterol levels compared with vehicle treatment and treatment with either GLP-1 or MK-801 (Fig. 1g). Furthermore, both GLP-1 monotherapy and GLP-1–MK-801 treatment resulted in decreased plasma triglyceride levels compared with the vehicle treatment (Fig. 1h). Notably, GLP-1–MK-801 also resulted in lower plasma triglycerides compared with MK-801 monotherapy. The effectiveness of GLP-1–MK-801 in regulating energy balance was further validated by its ability to normalize body weight and fat mass relative to age-matched control animals maintained on chow diet (Extended Data Fig. 3h–m). GLP-1–MK-801 also displayed superior weight loss efficacy compared with co-administration of GLP-1 and MK-801, despite having similar effects on food intake (Extended Data Fig. 3a–c). This implies that GLP-1–MK-801 reduces body weight through effects on both energy intake and energy expenditure. This notion was confirmed using metabolic cages, revealing that GLP-1–MK-801 counteracted the decrease in energy expenditure that is engaged by calorie-restricted mammals as an adaptive ‘starvation response’ to protect the organism from an excessive loss of body weight and fat mass (Fig. 1i–p and Extended Data Fig. 3n). Accordingly, despite losing 25% of their body mass, GLP-1–MK-801-treated mice maintained an energy expenditure that was similar to that of vehicle-treated control mice and was therefore also significantly higher than the gradually decreasing energy expenditure observed in mice that were calorie-restricted to match the weight loss trajectory of GLP-1–MK-801-treated mice. A subsequent study supported this finding by demonstrating that the energy expenditure of mice treated with the conjugate resembles that of the heavier control animals undergoing monotherapy treatment with GLP-1 and MK-801 (Extended Data Fig. 3o–s). This study also showed that GLP-1 and the GLP-1–MK-801 conjugate promote enhanced whole-body lipid oxidation, evident by a decrease in respiratory exchange ratio (RER) compared with vehicle or MK-801 treatment (Extended Data Fig. 3q,r).

**Extended Data Fig. 4:**
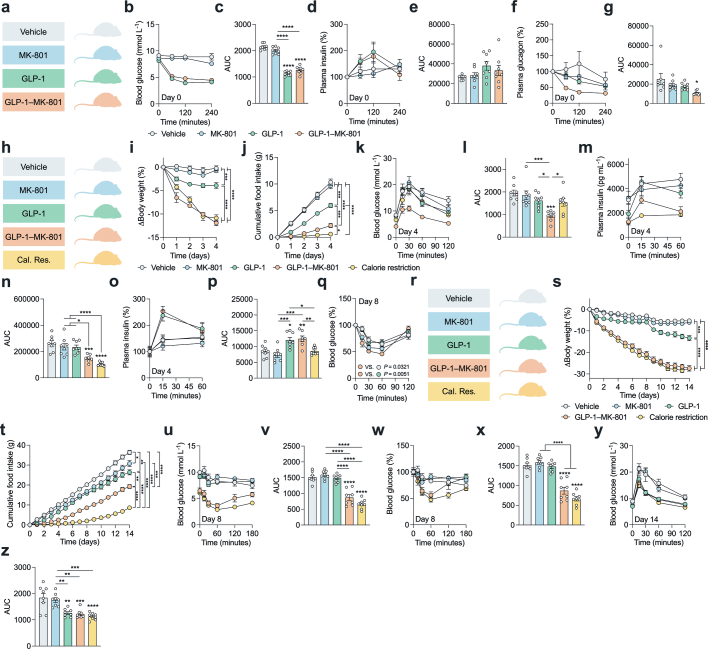
Evaluation of glucometabolic effects of GLP-1–MK-801. **a**-**g**, Compound tolerance test in response to a single s.c. injection of 100 nmol kg^−1^ MK-801 (*n* = 8 mice), 100 nmol kg^−1^ GLP-1 (*n* = 8 mice), 100 nmol kg^−1^, GLP-1–MK-801 (*n* = 8 mice), or vehicle (isotonic saline, *n* = 8 mice) in DIO mice. **a**, Schematic. **b**, Compound tolerance test on day 0. **c**, AUC of **b**. **d**, Plasma insulin levels during compound tolerance test. One mouse and one datapoint in vehicle group was omitted from analysis as they had insulin levels outside the assay range. **e**, AUC of **d**. **f**, Plasma glucagon levels during compound tolerance test. One mouse in vehicle group had supraphysiological glucagon levels outside the assay range and was omitted from analysis. **g**, AUC of **f**. **h**-**p**, Glucose-stimulated insulin secretion. Treatment of DIO mice with once-daily s.c. injections of 100 nmol kg^−1^ MK-801 (*n* = 8 mice and *n* = 4 cages), 100 nmol kg^−1^ GLP-1 (*n* = 8 mice and *n* = 4 cages), 100 nmol kg^−1^ GLP-1–MK-801 (*n* = 8 mice and *n* = 4 cages), calorie restriction (Cal. Res.) to match the weight loss of GLP-1–MK-801 (*n* = 8 mice and *n* = 5 cages) or vehicle (isotonic saline, *n* = 8 mice and *n* = 5 cages) for 4 days. On day 4, an intraperitoneal glucose tolerance test was conducted with collection of tail blood at timepoints 0, 15 and 60 min. **h**, Schematic. **i**, Change in body weight. **j**, Cumulative food intake. **k**, Glucose tolerance test on day 4. **l**, AUC of **k**. **m**, Glucose-stimulated insulin secretion. One datapoint in vehicle group, one datapoint in MK-801 group and one mouse and one datapoint in GLP-1–MK-801 group were outside assay range and omitted from analysis. **n**, AUC of **m**. **o**, Percentage to baseline of **m**. **p**, AUC of **o**. **q**, Percentage to baseline of insulin tolerance test in Fig. 1v. **r**-**z**, Insulin and glucose tolerance compared to mice undergoing calorie restriction to match the weight loss trajectory of GLP-1–MK-801. Treatment of DIO mice with once daily s.c. injections of 100 nmol kg^−1^ MK-801 (*n* = 8 mice and *n* = 4 cages), 100 nmol kg^−1^ GLP-1 (*n* = 8 mice and *n* = 4 cages), 100 nmol kg^−1^ GLP-1–MK-801 (*n* = 8 mice and *n* = 4 cages), calorie restriction (Cal. Res.) to match the weight loss of GLP-1–MK-801 (*n* = 8 mice and *n* = 4 cages) or vehicle (isotonic saline, *n* = 8 mice and *n* = 3-4 cages) for 14 days. On day 8, an intraperitoneal insulin tolerance test was conducted and on day 14 an intraperitoneal glucose tolerance test was conducted. **r**, Schematic. **s**, Change in body weight. **t**, Cumulative food intake. Four datapoints were excluded for one cage in vehicle group due to food spillage on day 11-14. **u**, Insulin tolerance test on day 8. **v**, AUC of **u**. **w**, Percentage to baseline of **u**. **x**, AUC of **w**. **y**, Glucose tolerance test on day 14. **z**, AUC of **y**. Data analysed by one-way ANOVA, multiple comparison, Bonferroni *post hoc* test (**c**, **e**, **g**, **l**, **n**, **p**, **v**, **x** and **z**) and two-way repeated measures ANOVA to assess main effects of treatment (**i**, **j**, **q**, **s** and **t**). Data represents mean ± SEM; ^*^P < 0.05, ^**^P < 0.01, ^***^P < 0.001, ^****^P < 0.0001. Detailed statistics are in Supplementary Table 1. The diagrams in **a**, **h** and **r** were created using BioRender. Source Data

Both MK-801 and GLP-1 have been linked to improvements in glucose homeostasis in mice^20,21^. Here we show that a single administration of GLP-1 or GLP-1–MK-801 elicits comparable reductions in the blood glucose levels in DIO mice, whereas equimolar MK-801 monotherapy does not acutely affect glycaemia (Fig. 1q–s and Extended Data Fig. 4a–c). The decrease in hyperglycaemia after administration of GLP-1 or GLP-1–MK-801 was not associated with changes in plasma insulin levels (Extended Data Fig. 4d, e). However, in comparison to vehicle-treated mice, mice treated with GLP-1–MK-801 demonstrated a slight decrease in plasma glucagon levels (Extended Data Fig. 4f,g). After 4 days of treatment, GLP-1 monotherapy and GLP-1–MK-801 displayed comparable benefits on glucose tolerance relative to vehicle treatment or treatment with MK-801 (Fig. 1t,u). In a follow-up study, a calorie-restricted weight-matched group was introduced alongside the vehicle, GLP-1, MK-801 and GLP-1–MK-801 groups (Extended Data Fig. 4h–j). In this study, the GLP-1–MK-801-treated cohort exhibited significantly enhanced glucose tolerance compared with all of the other groups (Extended Data Fig. 4k,l). Insulin levels during the glucose tolerance test were substantially lowered in the calorie-restricted mice and in mice treated with GLP-1–MK-801 (Extended Data Fig. 4m,n). However, only the animals that were administered with the incretin-based compounds (GLP-1 and GLP-1–MK-801) displayed increased baseline-corrected glucose-stimulated insulin secretion (Extended Data Fig. 4o,p). Mice that were treated with GLP-1–MK-801 for 8 days displayed improved insulin sensitivity as assessed by an insulin tolerance test compared with mice that were treated with either monotherapy or vehicle (Fig. 1v,w and Extended Data Fig. 4q). The improved insulin sensitivity after GLP-1–MK-801 treatment was confirmed in a 2-week study that, in addition to the pharmacological groups, included a calorie-restricted group weight-matched to the GLP-1–MK-801 group (Extended Data Fig. 4r–t). In this study, both the GLP-1–MK-801-treated mice and the calorie-restricted mice exhibited improved insulin sensitivity relative to the vehicle treatment and both monotherapy control groups (Extended Data Fig. 4u–x). After 2 weeks of treatment, mice treated with GLP-1–MK-801, as well as those subjected to calorie restriction or GLP-1 monotherapy, demonstrated enhanced glucose tolerance compared with mice that were treated with the vehicle or MK-801 monotherapy (Extended Data Fig. 4y,z). In summary, the favourable effects of GLP-1–MK-801 on glucose homeostasis seem to stem from both its incretin action and its weight-reducing effect.

## Safety profiling of GLP-1–MK-801

**Extended Data Fig. 5:**
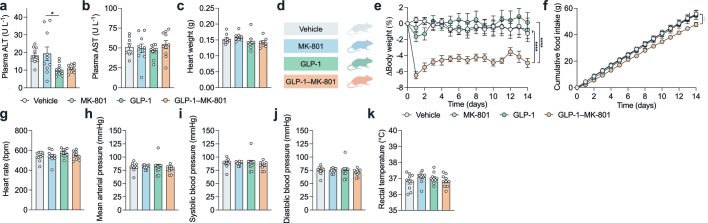
GLP-1–MK-801 is devoid of hallmark MK-801-induced adverse effects. **a**, **b**, Supporting data to study in Fig. 1a–h (vehicle (isotonic saline), *n* = 10 mice, 100 nmol kg^−1^ MK-801 *n* = 10 mice, 100 nmol kg^−1^ GLP-1 *n* = 10 mice, 100 nmol kg^−1^ GLP-1–MK-801 *n* = 10 mice). **a**, Plasma alanine transaminase (ALT) activity. **b**, Plasma aspartate aminotransferase (AST) activity. Two samples were removed from vehicle group, i.e., one sample was non-detectable and for another sample the activity was a significant outlier (Grubbs test). **c**, Heart weights of mice in Extended Data Figs. 3d–g and 3o–s (vehicle *n* = 8 mice, 100 nmol kg^−1^ MK-801 *n* = 8 mice, 100 nmol kg^−1^ GLP-1 *n* = 8 mice, 100 nmol kg^−1^ GLP-1–MK-801 *n* = 8 mice). **d**-**j**, Assessment of cardiovascular safety following chronic treatment of chow-fed lean C57BL/6 J mice with once-daily s.c. injections of 100 nmol kg^−1^ MK-801 (*n* = 9 mice and 4 cages), 100 nmol kg^−1^ GLP-1 (*n* = 10 mice and 4 cages), 100 nmol kg^−1^ GLP-1–MK-801 (*n* = 10 mice and 4 cages), or vehicle (isotonic saline, *n* = 10 mice and 4 cages) for 14 days. On day 14, mice were anesthetized, and blood pressure and echocardiograms were recorded. One mouse was euthanized on day 12 in MK-801 group due to sickness. **d**, Schematic. **e**, Change in body weight. **f**, Cumulative food intake. **g**, Heart rate in beats per minute (bpm). **h**, Mean arterial pressure. **i**, Systolic blood pressure. **j**, Diastolic blood pressure. **k**, Rectal temperature on day 14 of mice from study in Fig. 1a–h. Data analysed by one-way ANOVA, multiple comparison, Bonferroni *post hoc* test (**a**-**c** and **g**-**k**) and two-way repeated measures ANOVA to assess main effects of treatment (**e** and **f**). Data represents mean ± SEM; ^*^P < 0.05, ^**^P < 0.01, ^****^P < 0.0001. Detailed statistics are in Supplementary Table 1. The diagram in **d** was created using BioRender. Source Data

To evaluate the cardiometabolic safety of the conjugate, key markers of liver damage and cardiovascular health were assessed. Whereas GLP-1 monotherapy slightly reduced plasma aspartate aminotransferase (AST) levels compared with those in MK-801-treated mice, GLP-1–MK-801 treatment did not affect plasma AST or alanine transaminase (ALT) levels (Extended Data Fig. 5a,b). In DIO mice that were treated for 14 days with vehicle, MK-801, GLP-1 or GLP-1–MK-801, heart weights remained unaffected (Extended Data Fig. 5c). Subsequent haemodynamic assessments did not show any adverse effects of any of the treatments on heart rate or arterial blood pressure in lean mice receiving treatment for 14 days (Extended Data Fig. 5d–j).

To examine whether the chemical conjugation of MK-801 to GLP-1 mitigates the adverse hyperthermic and hyperlocomotive effects of MK-801, mice were subjected to body temperature measurements and open-field behavioural tests after chronic and acute drug administration, respectively. These experiments demonstrated that the relatively low dose of MK-801, required for achieving a synergistic weight loss by the conjugate, does not affect the body temperature after 14 days of repeated dosing, as none of the treatment groups displayed different core temperature compared with the vehicle-treated mice (Extended Data Fig. 5k). Finally, we demonstrated that the coupling of MK-801 to GLP-1 effectively eliminates the unfavourable hyperlocomotive reaction to MK-801 in an open-field test (Fig. 1x,y). Conversely, animals administered either GLP-1 or GLP-1–MK-801 exhibited diminished locomotion compared with both MK-801-treated and vehicle-treated mice.

## Pharmacological characterization

**Fig. 2:**
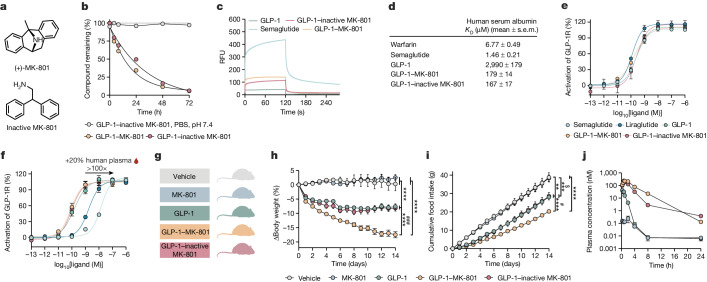
Pharmacokinetic assessments of GLP-1–MK-801. **a**, The chemical structures of (+)-MK-801 and 2,2-diphenylethan-1-amine (inactive MK-801). **b**, In vitro stability assay of GLP-1–MK-801 (*n* = 3) and GLP-1–inactive MK-801 (*n* = 3) incubated in human plasma at 37 °C and GLP-1–MK-801 (*n* = 1) incubated in PBS buffer, pH 7.4 at 37 °C. **c**,**d**, The interactions with human serum albumin of GLP-1, GLP-1–MK-801, GLP-1–inactive MK-801, semaglutide and warfarin were analysed using surface plasmon resonance (*n* = 3 per compound). **c**, Sensorgrams measured at 25 µM. RFU, relative fluorescence units. **d**, Dissociation constants were derived using a multi-site fit model. **e**,**f**, Dose–response curves for in vitro GLP-1 receptor activation of GLP-1, GLP-1–MK-801, GLP-1–inactive MK-801, liraglutide and semaglutide (*n* = 3 per compound). **e**, Dose–response curves. **f**, Dose–response curves in the presence of 20% human plasma. **g**–**i**, DIO mice were treated once-daily with s.c. injections of MK-801, GLP-1, GLP-1–inactive MK-801 or vehicle for 14 days. *n* = 8 mice. 100 nmol kg^−1^ dose. **g**, Schematic. **h**, Change in body weight. **i**, Cumulative food intake. **j**, The plasma concentration of MK-801, GLP-1, GLP-1–inactive MK-801 and GLP-1–MK-801 in chow-fed male C57BL/6J mice. *n* = 4 mice per group. 100 nmol kg^−1^ dose. Data were analysed using two-way repeated-measures ANOVA to assess main effects of treatment (**h**–**j**). Dissociation constants were determined using a multi-site fit model (**d**). Data are mean ± s.e.m. *P < 0.05, **P < 0.01, ***P < 0.001, ****P < 0.0001.^ #^denotes comparison between GLP-1–MK-801 and GLP-1–inactive MK-801. ^$^denotes comparison between vehicle and GLP-1–inactive MK-801. Detailed statistics are provided in Supplementary Table 1. The diagram in **g** was created using BioRender. Source Data

To examine whether the potent weight loss observed in vivo after GLP-1–MK-801 treatment is attributable to pharmacological synergy between GLP-1 and MK-801 or consequential to altered pharmacokinetic properties of the parent GLP-1 analogue, we designed and synthesized a conjugate comprising an inactive MK-801 surrogate^22^ (Supplementary Fig. 1). The inactivated conjugate displayed comparable plasma stability and human serum albumin (HSA) binding properties to GLP-1–MK-801 (Fig. 2a–d). These findings were corroborated by in vitro experiments showing similar GLP-1 receptor signalling properties of GLP-1–MK-801 and GLP-1–inactive MK-801 compared with lipidated analogues of GLP-1, semaglutide and liraglutide, and the parent GLP-1 analogue (Fig. 2e). However, in the presence of 20% human plasma, a 100-fold rightward curve shift was observed for the lipidated compounds, but not the conjugates and the GLP-1 analogue, indicating that MK-801 and inactive MK-801 do not associate strongly with HSA (Fig. 2f). Having demonstrated that GLP-1–MK-801 and GLP-1–inactive MK-801 display comparable pharmacokinetic properties in vitro, we next assessed their respective in vivo efficacies against the parent GLP-1 analogue in DIO mice. Treatment with GLP-1–inactive MK-801 produced no additional weight loss efficacy relative to GLP-1 monotherapy (Fig. 2g–i), indicating that the pronounced weight loss induced by GLP-1–MK-801 is driven by concerted and site-directed pharmacological GLP-1 receptor agonism and NMDA receptor antagonism. Finally, using liquid chromatography coupled with mass spectrometry (LC–MS), we quantified the plasma concentrations of GLP-1, GLP-1–inactive MK-801 and GLP-1–MK-801 after s.c. administration and observed a half-life of 1.9 h for GLP-1–MK-801, which is longer than the parent GLP-1 analogue, but comparable to that of the GLP-1–inactive MK-801 conjugate (Fig. 2j).

**Extended Data Fig. 6:**
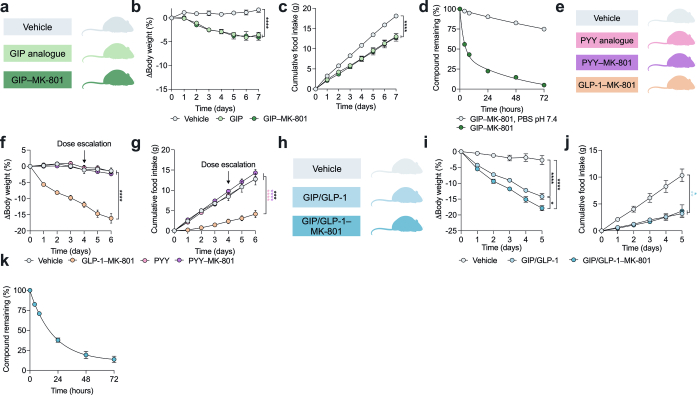
Assessment of MK-801 as a half-life extender of gut peptide hormones. **a**-**c**, Treatment of DIO mice with once-daily s.c. injections of 100 nmol kg^−1^ GIP analogue (*n* = 8 mice and *n* = 4 cages), 100 nmol kg^−1^ GIP–MK-801 (*n* = 8 mice and *n* = 4 cages) or vehicle (isotonic saline, *n* = 8 mice and *n* = 4 cages) for 7 days. **a**, Schematic. **b**, Change in body weight. **c**, Cumulative food intake. **d**, In vitro stability assay of GIP–MK-801 (*n* = 2) incubated in human plasma or PBS, pH = 7.4 (*n* = 1). **e**-**g**, Treatment of DIO mice with once daily s.c. injections of 100 nmol kg^−1^ PYY analogue (*n* = 7 mice and *n* = 6 cages), 100 nmol kg^−1^ PYY–MK-801 (*n* = 7 mice and *n* = 4 cages), 100 nmol kg^−1^ GLP-1–MK-801 (*n* = 7 mice and *n* = 5 cages) or vehicle (isotonic saline, *n* = 7 mice and *n* = 4 cages) for 6 days. The dose of PYY-based compounds was escalated at day 4 from 100 nmol kg^−1^ to 500 nmol kg^−1^. **e**, Schematic. **f**, Change in body weight. **g**, Cumulative food intake. **h**-**j**, Treatment of DIO mice with once daily s.c. injections of 50 nmol kg^−1^ GIP/GLP-1 (*n* = 6 mice and *n* = 3 cages), 50 nmol kg^−1^ GIP/GLP-1–MK-801 (*n* = 6 mice and *n* = 3 cages) or vehicle (isotonic saline, *n* = 6 mice and *n* = 3 cages) for 5 days. **h**, Schematic. **i**, Change in body weight. **j**, Cumulative food intake. **k**, In vitro stability assay of GIP/GLP-1–MK-801 (*n* = 3) incubated in human plasma. Data analysed by two-way repeated measures ANOVA to assess main effects of treatment (**b**, **c**, **f**, **g**, **i** and **j**). Data represents mean ± SEM; ^*^P < 0.05, ^****^P < 0.0001. Detailed statistics are in Supplementary Table 1 and chemical structures are in Supplementary Fig. 1. The diagrams in **a**, **e** and **h** were created using BioRender. Source Data

To further examine the possibility that MK-801 could convey protraction, we synthesized a series of conjugates comprising MK-801 in combination with other peptide analogues, peptide YY (PYY), glucose insulinotropic peptide (GIP) as well as a GIP/GLP-1 co-agonist, for which half-life extension has been shown to enhance weight loss efficacy^23–26^. Treatment of DIO mice with PYY–MK-801 and GIP–MK-801 did not affect body weight or food intake beyond that of the parent peptide analogues (Extended Data Fig. 6a–g and Supplementary Fig. 1). GIP/GLP-1 co-agonists, developed by merging the respective peptide sequences, potently lower body weight in rodents and humans^27,28^. Chemical synthesis and subsequent in vivo evaluation of a GIP/GLP-1–MK-801 co-agonist conjugate revealed enhanced weight loss in DIO mice compared with mice treated with the GIP/GLP-1 co-agonist alone (Extended Data Fig. 6h–k). These experiments support the notion that MK-801 does not enhance the pharmacokinetics of gut peptides and that the additional metabolic benefits and synergistic weight loss effects brought about by MK-801 are dependent on NMDA receptor antagonism in GLP-1-receptor-positive neurons.

## Neuronal effects of GLP-1–MK-801

**Fig. 3:**
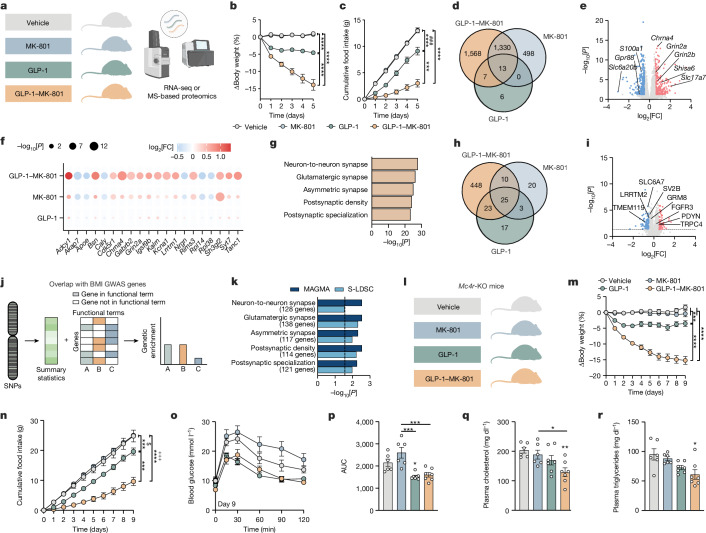
Effects of GLP-1–MK-801 on hypothalamic signalling. **a**–**g**,**j**,**k**, RNA-seq analysis of hypothalami from DIO mice treated once daily with s.c. injections of MK-801, GLP-1, GLP-1–MK-801 or vehicle for 5 days. *n* = 8 mice per group. 100 nmol kg^−1^ dose. **a**, Schematic. **b**, Change in body weight. **c**, Cumulative food intake. **d**, Venn diagram of differentially expressed genes. **e**, Volcano plot of differentially expressed genes in response to GLP-1–MK-801. FC, fold change. **f**, The top 20 differentially expressed genes found in the top five functional terms in **g**. **g**, The top five functional terms in response to GLP-1–MK-801 in the hypothalamus. **h**,**i**, MS-based proteomic analyses of hypothalami from DIO mice treated with once-daily s.c. injections of MK-801, GLP-1, GLP-1–MK-801 or vehicle for 5 days. *n* = 8 mice per group. 100 nmol kg^−1^ dose. **h**, Venn diagram of differentially expressed proteins. **i**, Volcano plot of proteins regulated in response to GLP-1–MK-801. **j**, Schematic of BMI GWAS integration. Differentially expressed proteins found in the top five functional terms were integrated with human BMI GWAS data. SNP, single-nucleotide polymorphism. **k**, Overlap analyses using the MAGMA and S-LDSC tools to compute BMI GWAS integration. **l**–**r**, Treatment of *Mc4r*-KO mice with once-daily s.c. administration of MK-801, GLP-1, GLP-1–MK-801 or vehicle for 9 days. *n* = 6–7 mice. 100 nmol kg^−1^ dose. One mouse in the MK-801 group had to be euthanized after the intraperitoneal glucose tolerance test. **l**, Schematic. **m**, Change in body weight. **n**, Cumulative food intake. **o**, Glucose tolerance test on day 9. **p**, AUC of data in **o**. **q**, Plasma cholesterol. **r**, Plasma triglycerides. Data were analysed using one-way ANOVA with Bonferroni post hoc multiple-comparison test (**p**–**r**) and two-way repeated-measures ANOVA to assess main effects of treatment (**b**, **c**, **m** and **n**). Data are mean ± s.e.m. *P < 0.05, **P < 0.01, ***P < 0.001, ****P < 0.0001. ^#^denotes comparison between MK-801 and GLP-1.^$^denotes comparison between vehicle and GLP-1. ^+^denotes comparison between vehicle and GLP-1–MK-801. Detailed statistics are provided in Supplementary Table 1. The diagrams in **a**, **d**, **h**, **j** and **l** were created using BioRender. Source Data

**Extended Data Fig. 7:**
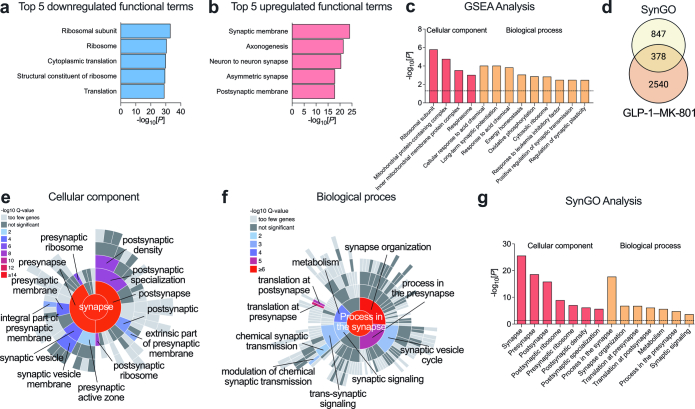
Bulk hypothalamic RNA sequencing data. **a**-**g**, Supplementary analyses of RNA sequencing data shown in Fig. 3a–g, j, k. **a**, Top 5 most enriched functional terms for the 1417 differentially expressed genes that were downregulated following GLP-1–MK-801 treatment. **b**, Top 5 enriched functional terms for the 1501 differentially expressed genes that were upregulated following GLP-1–MK-801 treatment. **c**, Top four cellular component and top nine biological process functional terms identified using gene set enrichment (GSEA) analysis. **d**, Venn diagram showing the overlap between the SynGO database and the 2918 uniquely differentially expressed genes in response to treatment with GLP-1–MK-801. **e**, Sunburst plot of differentially expressed genes belonging to cellular component functional terms computed with SynGO. Enrichment is colour coded by Q value for the functional terms. All level terms identified have been labelled. **f**, Sunburst plot of differentially expressed genes belonging to biological process functional terms computed with SynGO. Enrichment is colour coded by Q value for the functional terms. All level terms identified have been labelled. **g**, Top seven enriched functional terms for cellular component and top 7 functional terms enriched for biological process identified by SynGO. Benjamini-Hochberg adjusted *P* values were used for all analyses (**a**–**g**). The diagram in **d** was created using BioRender. Source Data

**Extended Data Fig. 8:**
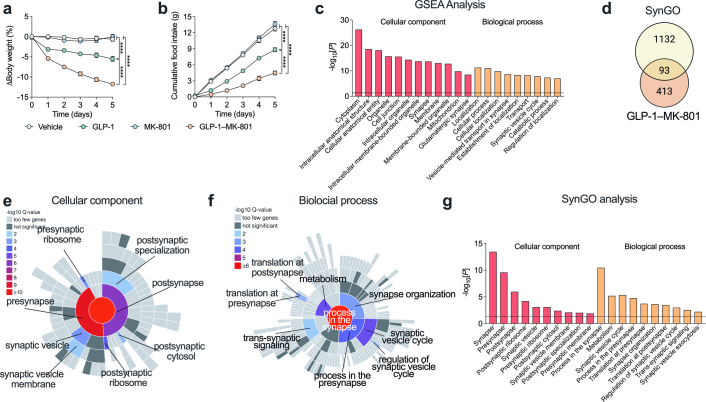
Mass spectrometry-based proteomics analyses of hypothalamic nuclei in response to GLP-1–MK-801. **a**-**g**, Proteomics data from study in Fig. 3h, i of mice treated with 100 nmol kg^−1^ MK-801 (*n* = 8 mice and *n* = 4 cages), 100 nmol kg^−1^ GLP-1 (*n* = 8 mice and *n* = 4 cages), 100 nmol kg^−1^ GLP-1–MK-801 (*n* = 8 mice and *n* = 4 cages) or vehicle (isotonic saline, *n* = 8 mice and *n* = 4 cages) for 5 days. **a**, Change in body weight. **b**, Cumulative food intake. **c**, Top twelve cellular component and top nine biological process ontologies identified using gene set enrichment (GSEA) analysis. **d**, Venn diagram showing the overlap between the SynGO database and the 506 uniquely differentially expressed proteins in response to GLP-1–MK-801. **e**, Sunburst plot of differentially expressed proteins belonging to cellular component functional terms computed with SynGO. Enrichment is colour coded by Q value for the functional terms. All level terms identified have been labelled. **f**, Sunburst plot of differentially expressed proteins belonging to biological process functional terms computed with SynGO. Enrichment is colour coded by Q value for the functional terms. All level terms identified have been labelled. **g**, Top ten SynGO enriched functional terms for cellular component and biological process. Data analysed by two-way repeated measures ANOVA to assess main effects of treatment (**a** and **b**). Benjamini-Hochberg corrected *P* (**c**-**g**) Data represents mean ± SEM; ^****^P < 0.0001. Detailed statistics are in Supplementary Table 1. The diagram in **d** was created using BioRender. Source Data

To understand the biological underpinnings of the metabolic benefits of GLP-1–MK-801, we performed RNA sequencing (RNA-seq) and MS-based proteomics analyses on hypothalami from DIO mice treated with vehicle, GLP-1, MK-801 or the GLP-1–MK-801 conjugate for 5 days (Fig. 3a–c and Extended Data Fig. 8a,b). A pronounced overlap in transcriptional signatures between GLP-1–MK-801 and each of GLP-1 and MK-801 was observed (Fig. 3d), suggesting that GLP-1–MK-801 engages signalling pathways that are related to both receptor systems in the hypothalamus. Notably, 1,568 transcripts were uniquely regulated in response to GLP-1–MK-801 treatment, emphasizing that coordinated activity between the two receptor targets in vivo potently influences hypothalamic transcription. Volcano plot visualization of GLP-1–MK-801 relative to vehicle revealed transcripts associated with glutamatergic signalling, such as *Grin2a*, *Grin2b*, *Shisa6* and *Slc17a7* among the most differentially upregulated genes (Fig. 3e). The targeted delivery of MK-801 was further emphasized by analysis of the top 20 differentially expressed genes derived from the top five enriched functional terms showing a similar but stronger transcriptional regulation for GLP-1–MK-801 in comparison to MK-801 (Fig. 3f). The transcriptional signatures identified in GLP-1–MK-801-treated animals were enriched for functional terms related to synaptic transmission, such as postsynaptic density and glutamatergic synapse (Fig. 3g and Extended Data Fig. 7a–g). These findings were substantiated by MS-based proteomics analyses in which we observed a more pronounced response to treatment with GLP-1–MK-801 compared with either monotherapy (Fig. 3h,i and Extended Data Fig. 8a,b). Moreover, the alterations induced by the conjugate in the hypothalamic proteome primarily highlight shifts in functional terms associated with cellular processes and synaptic function (Extended Data Fig. 8c–g). Collectively, these observations underscore that the pronounced weight loss caused by GLP-1–MK-801 coincides with changes in hypothalamic neuroplasticity and glutamatergic signalling. Thus, the conjugate seemingly harnesses the dual pharmacological benefits of hypothalamic GLP-1 receptor agonism and NMDA receptor antagonism while mitigating adverse behavioural effects linked to the latter. To probe the translational potential of GLP-1–MK-801, we assessed whether the differentially expressed genes regulated in response to treatment overlapped with genes located in obesity-associated GWAS loci (Fig. 3j). Using two independent bioinformatic tools^29,30^, we found a significant overlap with transcripts associated with genetic susceptibility to obesity in humans, suggesting that GLP-1–MK-801 targets biological pathways implicated in common polygenic forms of human obesity^1,31^ (Fig. 3k).

Weight loss drugs targeting the hypothalamus often converge on the leptin–melanocortin pathway. To assess whether the anti-obesity properties of GLP-1–MK-801 are dependent on this key energy balance-governing pathway, we tested the weight loss efficacy in DIO *Mc4r*-KO (also known as melanocortin-4 receptor) mice. We observed a pronounced vehicle-corrected weight loss of 15.2% in the group treated with GLP-1–MK-801 compared with 3.5% in the group treated with the parent GLP-1 analogue after 9 days of treatment, underscoring that the weight-lowering efficacy of the conjugate is intact in the absence of functional MC4R signalling (Fig. 3l,m). The weight loss was accompanied by a decrease in food intake in comparison to mice treated with GLP-1 or MK-801 monotherapy or vehicle (Fig. 3n). Despite the large differences in weight loss between GLP-1 and GLP-1–MK-801, the two compounds exhibited similar benefits on glucose tolerance in *Mc4r*-KO mice. While only the GLP-1-treated group achieved statistical significance relative to the vehicle group, both the GLP-1 and GLP-1–MK-801 groups exhibited significantly enhanced glucose tolerance in comparison to animals treated with MK-801 (Fig. 3o,p). By contrast, only GLP-1–MK-801 treatment lowered plasma cholesterol and plasma triglycerides as compared to vehicle or MK-801 treatment (Fig. 3q,r). These findings emphasize that targeted antagonism of NMDA receptor signalling lowers body weight independently of MC4R, indicating that this preclinical drug candidate might not only be effective in treating polygenic obesity but also holds potential as an effective weight loss strategy for patients with loss-of-function mutations in *MC4R*, the most common form of monogenic obesity^32^.

## GLP-1–MK-801 versus semaglutide

**Fig. 4:**
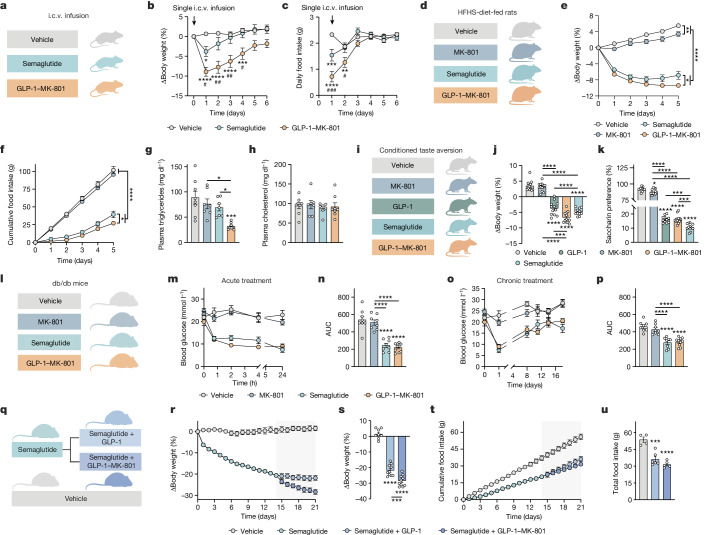
GLP-1–MK-801 versus semaglutide in preclinical models of obesity and diabetes. **a**–**c**, i.c.v. infusions of 0.22 nmol semaglutide (*n* = 15 mice), 0.22 nmol GLP-1–MK-801 (*n* = 15 mice) or vehicle (*n* = 12 mice) in high-fat high-sucrose (HFHS)-fed mice. **a**, Schematic. **b**, Change in body weight. **c**, Daily food intake. One datapoint was excluded for one vehicle mouse owing to a measurement error. **d**–**h**, Once-daily s.c. treatment of Sprague-Dawley rats maintained on an HFHS diet for 4 weeks with MK-801 (100 nmol kg^−1^), semaglutide (10 nmol kg^−1^), GLP-1–MK-801 (100 nmol kg^−1^) or vehicle. *n* = 7–8 rats. **d**, Schematic. **e**, Change in body weight. **f**, Cumulative food intake. **g**, Plasma triglycerides. One plasma sample was lost in the semaglutide group. **h**, Plasma cholesterol. **i**–**k**, CTA analysis of chow-fed Wistar rats after treatment with MK-801 (100 nmol kg^−1^), GLP-1 (100 nmol kg^−1^), GLP-1–MK-801 (100 nmol kg^−1^), semaglutide (10 nmol kg^−1^) or vehicle. *n* = 12 rats per group. **i**, Schematic. **j**, Change in body weight 24 h after dosing. **k**, Saccharin preference. Saccharin intake data for one mouse in the GLP-1 group was not collected due to a sensor malfunction. **l**–**p**, Treatment of *db/db* mice with once-daily s.c. injections of MK-801 (100 nmol kg^−1^), semaglutide (10 nmol kg^−1^), GLP-1–MK-801 (100 nmol kg^−1^) or vehicle for 18 days. *n* = 8–10 mice per group. **l**, Schematic. **m**, Compound tolerance test on day 0. **n**, Area under the curve of data in **m**. **o**, Basal blood glucose. **p**, Area under the curve of data in **o**. **q**–**u**, DIO mice were treated once-daily with s.c. injections of semaglutide (*n* = 20 mice, 10 nmol kg^−1^) or vehicle (*n* = 8 mice). After 14 days of treatment, semaglutide-treated mice were randomized to receive semaglutide and GLP-1 (100 nmol kg^−1^) as co-administration or semaglutide and GLP-1–MK-801 (100 nmol kg^−1^) as co-administration. **q**, Schematic. **r**, Change in body weight. **s**, Change in body weight after 21 days. **t**, Cumulative food intake. **u**, Total food intake at day 21. Data were analysed using one-way ANOVA with Bonferroni post hoc multiple-comparison test (**g**, **h**, **j**, **k**, **n**, **p**, **s** and **u**), two-way repeated-measures ANOVA to assess the main effects of treatment (**e** and **f**) and two-way ANOVA with Bonferroni post hoc multiple-comparison test (**b** and **c**). Data are mean ± s.e.m. *P < 0.05, **P < 0.01, ***P < 0.001, ****P < 0.0001. ^#^*P* < 0.05, ^##^*P* < 0.01, ^###^*P* < 0.001 denotes comparison between GLP-1–MK-801 and semaglutide. Detailed statistics are provided in Supplementary Table 1. The diagrams in **a**, **d**, **i**, **l** and **q** were created using BioRender. Source Data

To further scrutinize the translational potential of GLP-1–MK-801, we benchmarked it against semaglutide, a pharmacokinetically optimized GLP-1 receptor agonist approved for treatment of obesity and clinically available as a once-weekly injectable^33^. To directly evaluate the head-to-head weight-lowering potential of semaglutide versus GLP-1–MK-801, we performed single intracerebroventricular (i.c.v.) infusions of equimolar concentrations of GLP-1–MK-801 or semaglutide (Fig. 4a). This study revealed a superior vehicle-corrected weight loss of 9.5% in response to the GLP-1–MK-801 infusion relative to a weight loss of 4.5% after semaglutide infusion (Fig. 4b). Notably, over the subsequent 6 days, we observed a sustained body-weight-lowering effect after infusion of GLP-1–MK-801, but not semaglutide, suggesting that the targeted inhibition of NMDA receptors in GLP-1 receptor-positive neurons might promote sustained changes to the defended level of adiposity. The enhanced weight loss observed in response to GLP-1–MK-801 coincided with a more pronounced decrease in food intake within the first 48 h after the infusion relative to semaglutide-treated mice (Fig. 4c). Together these data support a stronger brain dependence of the body-weight-lowering effects of GLP-1–MK-801 compared with semaglutide. At the same time, the data imply that a medicinal chemistry campaign focusing on engineering pharmacokinetically optimized GLP-1–MK-801 conjugates might give rise to drugs that could outperform the current incretin-based treatments for obesity, such as semaglutide and tirzepatide.

To substantiate the weight-lowering efficacy in another rodent species, Sprague-Dawley rats fed a high-fat high-sugar diet for 4 weeks were treated with once-daily s.c. injections of vehicle, MK-801, GLP-1–MK-801 or semaglutide at a dose that has previously been demonstrated to induce maximal body weight loss in rodents^34^ (Fig. 4d). After 5 days of treatment, rats treated with MK-801, semaglutide or GLP-1–MK-801 had significantly decreased body weight compared with the vehicle-treated rats. Semaglutide-treated rats exhibited a greater weight loss compared with MK-801-treated rats, and rats that were treated with GLP-1–MK-801 had significantly reduced body weight compared with rats that were treated with either MK-801 or semaglutide (Fig. 4e). Semaglutide and GLP-1–MK-801, but not MK-801 monotherapy, lowered food intake in rats, with GLP-1–MK-801 treatment resulting in a more pronounced reduction compared with semaglutide (Fig. 4f). Moreover, rats treated with GLP-1–MK-801 had reduced levels of circulating triglycerides compared with rats that were treated with either semaglutide or MK-801. No changes in plasma cholesterol levels were observed for any of the treatments (Fig. 4g,h).

**Extended Data Fig. 9:**
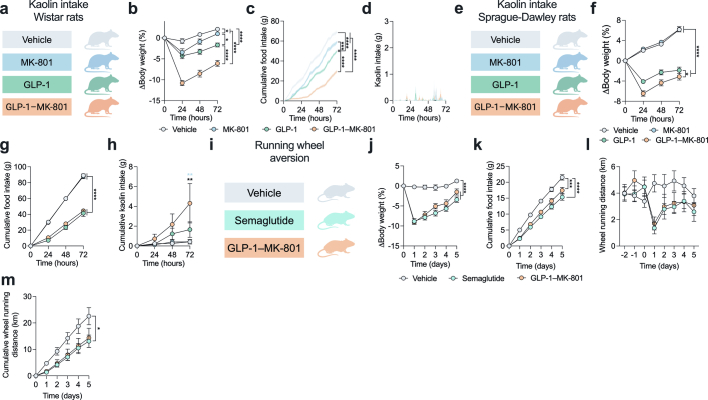
Assessment of aversion in rodents. **a**-**d**, Chow-fed Wistar rats were dosed once-daily with s.c. injections of 100 nmol kg^−1^ MK-801 (*n* = 12 rats), 100 nmol kg^−1^ GLP-1 (*n* = 12 rats), 100 nmol kg^−1^ GLP-1–MK-801 (*n* = 12 rats) or vehicle (isotonic saline, *n* = 13 rats) for three consecutive days while being offered the voluntary choice between chow and kaolin (a non-food product). **a**, Schematic. **b**, Change in body weight. **c**, Cumulative food intake. One food monitor did not work for one mouse in vehicle group. **d**, Time-resolved kaolin intake. One datapoint in GLP-1–MK-801 was omitted due to extensive food spillage. **e**-**h**, Chow-fed Sprague-Dawley rats were dosed once-daily with s.c. injections of 100 nmol kg^−1^ MK-801 (*n* = 8 rats), 100 nmol kg^−1^ GLP-1 (*n* = 8 rats), 100 nmol kg^−1^ GLP-1–MK-801 (*n* = 8 rats) or vehicle (isotonic saline, *n* = 8 rats) for three consecutive days while being offered the choice between chow and kaolin. **e**, Schematic. **f**, Change in body weight. **g**, Cumulative food intake. **h**, Cumulative kaolin intake. Two data points were omitted from analysis in vehicle group due to extensive food spillage. **i**-**m**, Wheel running study investigating aversive behaviour in response to treatment. Lean chow-fed male C57BL/6 J mice were single-housed in cages equipped with a running wheel and allowed 3 days of habituation before being treated with once-daily s.c. injections of 100 nmol kg^−1^ GLP-1–MK-801 (*n* = 8 mice), 10 nmol kg^−1^ semaglutide (*n* = 8 mice) or vehicle (isotonic saline, *n* = 7 mice) for 5 days with daily measurements of running distance at the time of injection. One mouse in vehicle group was excluded from analysis due to sickness. **i**, Schematic. **j**, Change in body weight. **k**, Cumulative food intake. **l**, Daily running wheel distance during habituation and treatment period. One datapoint in vehicle group was excluded due to the running wheel got stuck between day -1 to day 0. **m**, Cumulative running wheel distance during treatment period. Data analysed by two-way repeated measures ANOVA to assess main effect effects of treatment (**b**, **c**, **f**, **g**, **j**, **k** and **m**) and two-way ANOVA, multiple comparison, Bonferroni *post hoc* test (**h**). Data represents mean ± SEM; ^*^P < 0.05, ^**^P < 0.01, ^***^P < 0.001, ^****^P < 0.0001. Detailed statistics are in Supplementary Table 1. The diagrams in **a**, **e** and **i** were created using BioRender. Source Data

To further assess the adverse profile of GLP-1–MK-801, we performed a series of complementary studies in mice and rats. A conditioned-taste aversion (CTA) experiment in male chow-fed Wistar rats affirmed a potent weight loss in response to GLP-1, semaglutide and GLP-1–MK-801 compared with MK-801 and vehicle treatment. Rats that were treated with GLP-1–MK-801 exhibited a lower body weight compared with those that were treated with GLP-1 (Fig. 4i,j). Notably, although semaglutide resulted in a slightly less pronounced weight loss, it elicited a significantly greater aversive response compared with GLP-1–MK-801 (Fig. 4k). None of the tested treatments, including GLP-1–MK-801, triggered pica behaviour in Wistar rats, indicated by the low consumption of the non-nutritive substance kaolin (Extended Data Fig. 9a–d). However, in Sprague Dawley rats, GLP-1 monotherapy significantly increased kaolin intake compared with vehicle-treated rats and rats treated with MK-801 (Extended Data Fig. 9e–h). GLP-1–MK-801 did not elicit pica behaviour in Sprague Dawley rats. Together, these rat studies suggest that the weight-lowering potential of GLP-1–MK-801 is not associated with exacerbated nausea. Exercise avoidance behaviour is a sensitive indicator of substance-induced malaise in mice^35^. Here we demonstrate that mice treated with either semaglutide or GLP-1–MK-801 exhibit a similar yet transient effect on exercise avoidance (Extended Data Fig. 9i–m). This further underlines that the potent weight-reducing benefits of the bimodal compound are not linked to an aggravated adverse profile when compared to marketed incretin-based drugs such as semaglutide.

As NMDA receptors have previously been associated with regulation of glucose metabolism^20^, we evaluated the glucometabolic properties of GLP-1–MK-801 in the diabetic *db*/*db* mouse model. We observed that GLP-1–MK-801 and semaglutide elicited comparable glucose-lowering effects compared with vehicle and MK-801 monotherapy (Fig. 4l–p). Finally, to expand on the opportunity for NMDA receptor antagonism as a complementary pharmacological partner to GLP-1 receptor agonism, we pretreated DIO mice with semaglutide for 14 days and subsequently randomized the mice to stay on semaglutide in combination with either GLP-1–MK-801 or the GLP-1 monotherapy (Fig. 4q). Mice that received GLP-1–MK-801 on top of semaglutide displayed potentiated weight loss compared with mice that were co-treated with semaglutide and GLP-1 (Fig. 4r–u). Collectively, these data demonstrate that GLP-1–MK-801 corrects hypertriglyceridaemia and hyperglycaemia with improved or similar efficacy to semaglutide in animal models of obesity and type 2 diabetes. Moreover, the observation that adding GLP-1–MK-801 treatment on top of semaglutide leads to an additional weight loss of 7% in mice that have reached a weight loss plateau with semaglutide provides additional support for integrating NMDA receptor antagonism in next-generation weight loss therapeutics.

## Brain activity profiling of GLP-1–MK-801

**Fig. 5:**
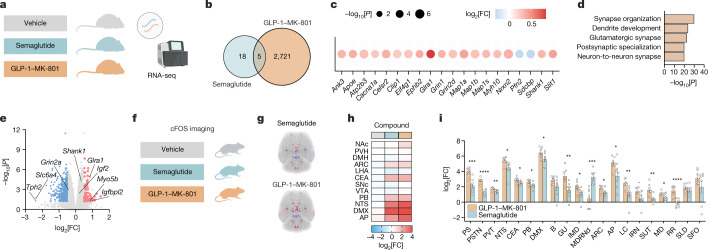
The effects of GLP-1–MK-801 on the hypothalamic transcriptome and whole-brain activity. **a**–**e**, RNA-seq analysis of hypothalami from DIO mice treated once-daily with s.c. injections of semaglutide (10 nmol kg^−1^), GLP-1–MK-801 (100 nmol kg^−1^) or vehicle for 5 days. *n* = 6 mice. **a**, Schematic. **b**, Venn diagram of differentially expressed genes. **c**, The top 20 most differentially expressed genes found in the top 5 functional terms in **d**. **d**, The top 5 functional terms between GLP-1–MK-801 and semaglutide treatments. **e**, Volcano plot of differentially expressed genes in response to GLP-1–MK-801 relative to semaglutide. **f**–**i**, Whole-brain 3D mapping and quantification of cFOS responses to a single s.c. injection with semaglutide (10 nmol kg^−1^), GLP-1–MK-801 (100 nmol kg^−1^) or vehicle in lean mice. *n* = 7–8 mice. One mouse in the GLP-1–MK-801 group was excluded due to sample-processing deviation. **f**, Schematic. **g**, Mouse brain images showing a heat map of the averaged changes in cFOS expression in response to treatments. **h**, Heat map of cFOS activity in brain regions associated with appetite regulation. **i**, Comparison of changes in cFOS expression in the top 20 most significantly regulated brain regions. Data were analysed using an unpaired two-tailed *t*-test (**i**). Data are mean ± s.e.m. *P < 0.05, **P < 0.01, ***P < 0.001, ****P < 0.0001. Definitions of brain region abbreviations are provided in the ‘cFOS whole-brain imaging’ section of the Methods. Detailed statistics are provided in Supplementary Table 1. The diagrams in **a**, **b** and **f** were created using BioRender. Source Data

**Extended Data Fig. 10:**
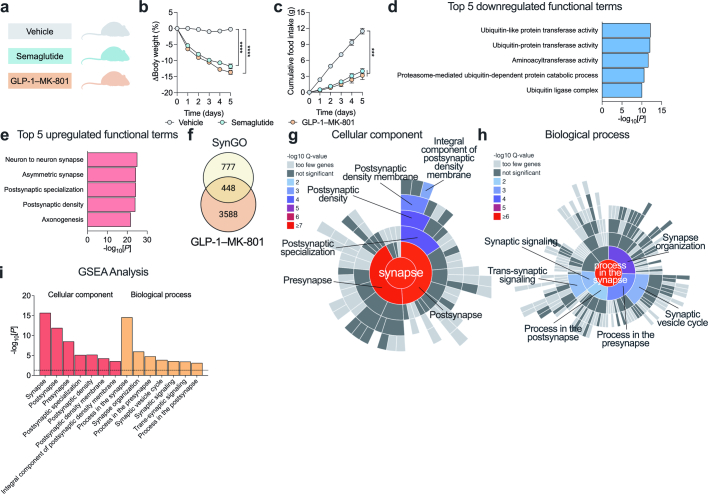
Bulk RNA-sequencing of hypothalamic nuclei comparing GLP-1–MK-801 and semaglutide treatments. **a**-**i**, Supporting data for study in Fig. 5a–e. Treatment of DIO mice with once-daily s.c. injections of 10 nmol kg^−1^ semaglutide (*n* = 6 mice and *n* = 3 cages), 100 nmol kg^−1^ GLP-1–MK-801 (*n* = 6 mice and *n* = 3 cages) or vehicle (isotonic saline, *n* = 6 mice and *n* = 3 cages) for 5 days. **a**, Schematic. **b**, Change in body weight. **c**, Cumulative food intake. **d**, Top five most enriched functional terms of the 2176 differentially expressed genes that were downregulated following GLP-1–MK-801 treatment relative to semaglutide. **e**, Top five most enriched functional terms for the 1866 differentially expressed genes that were upregulated following GLP-1–MK-801 treatment relative to semaglutide. **f**, Venn diagram showing the overlap between the SynGO database and the 4042 differentially expressed genes in response to GLP-1–MK-801 relative to semaglutide. **g**, Sunburst plot of differentially expressed genes belonging to cellular component functional terms computed with SynGO. Enrichment is colour coded by Q value for the functional terms. All level terms identified have been labelled. **h**, Sunburst plot of differentially expressed genes belonging to biological process functional terms computed with SynGO. Enrichment is colour coded by Q value for the functional terms. All level terms identified have been labelled. **i**, Top seven SynGO enriched functional terms for cellular component and biological process. Data analysed by two-way repeated measures ANOVA to assess main effects of treatment (**b** and **c**). Benjamini-Hochberg adjusted *P* (**d**-**i**). Data represents mean ± SEM; ^***^P < 0.001, ^****^P < 0.0001. Detailed statistics are in Supplementary Table 1. The diagrams in **a** and **f** were created using BioRender. Source Data

Encouraged by the transcriptomic data indicating that the weight loss is evoked through a mechanism of action involving the combined pharmacological actions of NMDA receptor antagonism and GLP-1 receptor agonism in the hypothalamus, we performed a comprehensive comparison between GLP-1–MK-801 and the long-acting GLP-1 receptor agonist, semaglutide. The substantial difference in weight loss between GLP-1–MK-801 treatment and the parent GLP-1 analogue could potentially confound the interpretation of differences in transcriptional regulation. To correct for this, we performed RNA-seq analysis of hypothalami obtained from animals treated with daily injections of either GLP-1–MK-801 or a dose of semaglutide that was equipotent in terms of weight-lowering efficacy over 5 days of treatment, that is, mice had achieved a comparable weight loss and reduction in food intake as that of GLP-1–MK-801-treated mice (Extended Data Fig. 10a–c). Similar to our previous RNA-seq analysis, we demonstrate that treatment with GLP-1–MK-801 significantly regulates around 150 times the number of hypothalamic transcripts relative to the weight-matched semaglutide-treated group, emphasizing a mode of action for GLP-1–MK-801 that involves biological pathways independent of the GLP-1 receptor signalling pathway (Fig. 5a,b). Further analyses of differentially expressed genes between GLP-1–MK-801 and semaglutide treatment groups support this notion by showing enrichment of functional terms pertaining to glutamatergic signalling and synaptic plasticity among the most upregulated genes. These observations were further supported by pathway analyses confirming the strong annotations related to NMDA receptor signalling (Fig. 5c–e and Extended Data Fig. 10d–i).

**Extended Data Fig. 11:**
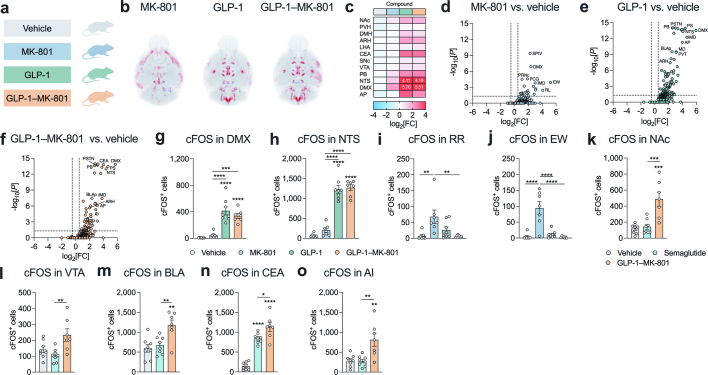
Whole brain cFos expression in response to semaglutide, MK-801, GLP-1 and GLP-1–MK-801. **a**-**j**, Whole-brain 3D mapping and quantification of cFOS responses to treatment single s.c. injection with 100 nmol kg^−1^ MK-801 (*n* = 7 mice), 100 nmol kg^−1^ GLP-1 (*n* = 8 mice), 100 nmol kg^−1^ GLP-1–MK-801 (*n* = 7 mice) or vehicle (isotonic saline, *n* = 8 mice) in lean mice. One mouse in MK-801 group, GLP-1–MK-801 group and vehicle group were excluded from analysis due to sample processing related deviation. **a**, Schematic. **b**, Mouse brain images showing heatmap of averaged changes in cFOS expression in response to treatments relative to vehicle. Red areas indicate increased cFOS activity and blue areas indicate decreased cFOS activity. **c**, Heatmap of cFOS activity in brain regions involved in body weight regulation. Values exceeding the colour scale are presented with the numeric values. **d**, Volcano plot of brain regions regulated in response to MK-801 relative to vehicle. Selected regions are labelled. **e**, Volcano plot of brain regions regulated in response to GLP-1 relative to vehicle. Selected regions are labelled. **f**, Volcano plot of brain regions regulated in response to GLP-1–MK-801 relative to vehicle. Selected regions are labelled. **g**, Quantification of cFOS^+^ cells in dorsal motor nucleus X (DMX). **h**, Quantification of cFOS^+^ cells in nucleus of the solitary tract (NTS). **i**, Quantification of cFOS^+^ cells in rostral linear nucleus raphe (RR). **j**, Quantification of cFOS^+^ cells in Edinger-Westphal nucleus (EW). **k**-**o**, cFOS^+^ whole-brain imaging data came from mice in Fig. 5f–i. One mouse in GLP-1–MK-801 group excluded due to sample processing related deviation. **k**, cFOS^+^ cells in nucleus accumbens (NAc). **l**, cFOS^+^ cells in ventral tegmental area (VTA). **m**, cFOS^+^ cells in basolateral amygdala nucleus (BLA). **n**, cFOS^+^ cells in central amygdala (CEA). **o**, cFOS^+^ cells in agranular insular area (AI). Data analysed by one-way ANOVA, multiple comparison, Bonferroni *post hoc* test (**g**-**o**). Data analysed by one-way ANOVA, multiple comparison, Bonferroni *post hoc* test (**g**-**o**). Benjamini-Hochberg adjusted *P* (**d**-**f**). Data represents mean ± SEM; ^**^P < 0.01, ^***^P < 0.001, ^****^P < 0.0001. For brain region abbreviations please refer to ‘cFos whole-brain imaging’ in the methods section. Detailed statistics are in Supplementary Table 1. The diagram in **a** was created using BioRender. Source Data

**Extended Data Fig. 12:**
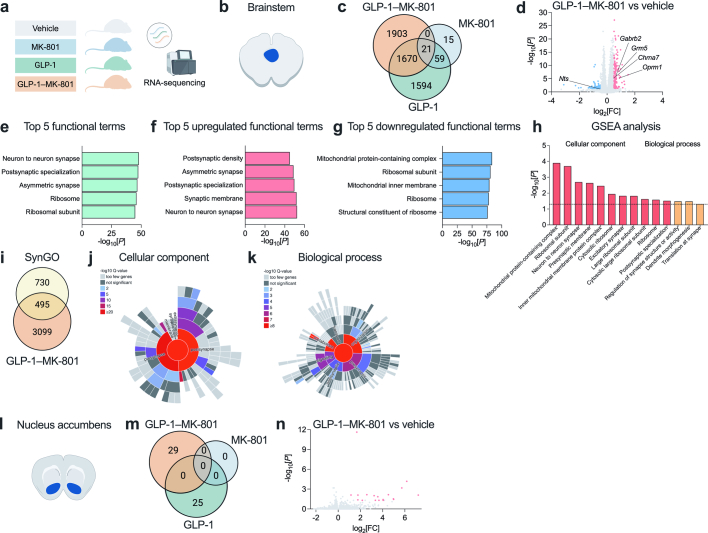
Bulk RNA sequencing of brainstem and nucleus accumbens after treatment with GLP-1–MK-801. **a**-**n**, Brain nuclei for bulk RNA sequencing of the brainstem came from mice in Fig. 3h, i and Extended Data Fig. 8 treated with 100 nmol kg^−1^ MK-801 (*n* = 8 mice), 100 nmol kg^−1^ GLP-1 (*n* = 8 mice), 100 nmol kg^−1^ GLP-1–MK-801 (*n* = 8 mice) or vehicle (isotonic saline, *n* = 8 mice) for 5 days. **a**, Schematic. **b**, Schematic highlighting the brainstem. **c**, Venn diagram of differentially expressed genes in response to treatments. **d**, Volcano plot for differentially expressed genes in response to GLP-1–MK-801. **e**, Top five most enriched functional terms for the 3594 differentially expressed genes between GLP-1–MK-801 and vehicle. **f**, Top five most enriched functional terms for the 2076 differentially expressed genes that were upregulated in response to GLP-1–MK-801 treatment relative to vehicle. **g**, Top five most enriched functional terms for the 1518 differentially expressed genes that were downregulated following GLP-1–MK-801 treatment relative to vehicle. **h**, Top eleven cellular component and top three biological process functional terms identified using gene set enrichment (GSEA) analysis. **i**, Venn diagram showing the overlap with the SynGO database and the 3594 differentially expressed genes in response to GLP-1–MK-801. **j**, Sunburst plot of differentially expressed genes belonging to cellular component functional terms computed with SynGO. Enrichment is colour coded by Q value for the functional terms. All level terms identified have been labelled. **k**, Sunburst plot of differentially expressed genes belonging to biological process functional terms computed with SynGO. Enrichment is colour coded by Q value for the functional terms. All level terms identified have been labelled. **l**, Schematic highlighting the nucleus accumbens. **m**, Venn diagram of differentially expressed genes in response to treatments. **n**, Volcano plot for differentially expressed genes of GLP-1–MK-801. Benjamini-Hochberg adjusted *P* (**c**-**n**). Detailed statistics are in Supplementary Table 1. The diagrams in **a**-**c**, **i**, **l** and **m** were created using BioRender. Source Data

GLP-1 and NMDA receptors are expressed throughout the mammalian brain and, although reports indicate that GLP-1 analogues engage central appetite circuits through distinct subpopulations of neurons (most notably in the brainstem and the hypothalamus)^36^, feedforward neural pathways modulated by inhibition of NMDA receptors specifically in GLP-1-receptor-positive neurons may differ from those engaged by exclusive activation of GLP-1 receptors. To understand whether the GLP-1–MK-801 conjugate activates distinct neural circuitries, we used light-sheet microscopy and cFOS immunohistochemistry to obtain quantitative 3D whole-brain activity signatures in response to GLP-1–MK-801 and semaglutide dosing (Fig. 5f,g). A large overlap in neuronal activity patterns was observed between the two treatments and, in agreement with previous research^37^, strong signals were detected in the arcuate nucleus, in the central amygdala and in subregions of the brainstem, including the area postrema, the nucleus of the solitary tract and the dorsal motor nucleus of the vagus nerve (Fig. 5h,i). The most noticeable difference in whole-brain activity signatures between GLP-1–MK-801 and semaglutide was the significantly higher number of cFOS^+^ cells in the nucleus accumbens in response to GLP-1–MK-801 compared with semaglutide and vehicle treatment. However, this might be consequential to the fatty acid sidechain^38^ and not related to NMDA receptor modulation (Extended Data Fig. 11a–j). Subsequent subregion analyses showed that the ventral tegmental area as well as subregions of the amygdala displayed enhanced activity patterns after GLP-1–MK-801 treatment (Extended Data Fig. 11k–o). Collectively, these analyses suggest that the mesolimbic reward system may be more potently engaged with GLP-1–MK-801 than semaglutide treatment. To gain deeper insights into the impact of the conjugate on both the brainstem and the mesolimbic reward system, we conducted complementary transcriptomics analyses (Extended Data Fig. 8a,b). Similar to the transcriptomic changes observed in the hypothalamus, we found that the conjugate prompted transcriptional patterns enriched for functional terms associated with synaptic transmission, including postsynaptic specialization and neuron-to-neuron synapse in the brainstem (Extended Data Fig. 12a–k). By contrast, substantial transcriptional alterations were not induced by the conjugate in the nucleus accumbens (Extended Data Fig. 12l–n). Together, these findings necessitate further investigations to ascertain whether coordinated inhibition of NMDA receptors in GLP-1 receptor neurons modulates neuronal activity beyond canonical feeding regions, such as the mesolimbic dopaminergic reward pathway.

## Discussion

Here we demonstrate the efficacy of peptide-targeted NMDA receptor inhibition for obesity treatment. To bypass the pharmacological challenges associated with unspecific blocking of NMDA receptors, we designed a peptide–drug conjugate comprising the NMDA receptor antagonist MK-801 and a GLP-1 peptide analogue. By using a reducible disulfide linker, we built in a redox-sensitive release mechanism to facilitate intracellular release of the unmodified NMDA receptor antagonist, enabling the combined cellular actions of GLP-1 receptor agonism and NMDA receptor antagonism.

Treatment with the GLP-1–MK-801 conjugate potently reverses obesity, diabetes and dyslipidaemia in a wide range of rodent models of obesity and metabolic disease. Although the exact mechanisms by which the coordinated actions of GLP-1 receptor agonism and NMDA receptor antagonism correct metabolic diseases await further elucidation, the extensive alterations in transcriptomic and proteomic responses within the hypothalamus, linked to glutamatergic signalling and synaptic plasticity, imply that the conjugate might elicit neurostructural changes in GLP-1-receptor-expressing neurons. This observation aligns with previous work showing that pharmacological inhibition or genetic ablation of NMDA receptors in agouti-related-protein (AgRP)-expressing neurons alters synaptic adaptation to fasting^39,40^. Moreover, previous consideration of NMDA receptor antagonism as potential treatment for binge-eating disorder suggests that the weight-lowering benefits linked to GLP-1–MK-801 could be the result of a combined impact on both energy homeostasis and disordered eating behaviours^10,14,41^.

Although our data align with the notion that inhibiting NMDA receptor signalling in the hypothalamus decreases food intake and body weight^8,40^, earlier studies have demonstrated that NMDA receptor antagonism in the brainstem attenuates vagus-mediated meal-related satiety^6,7,42^. The bidirectional effects of NMDA receptor antagonism on feeding could potentially diminish the weight-lowering efficacy of systemic exposure to NMDA receptor antagonists. Supported by the substantial weight-loss efficacy of the GLP-1–MK-801 conjugate and the potentiated hypothalamic changes in transcripts and proteins related to NMDA receptor-linked neuroplasticity, the alteration in MK-801 biodistribution governed by GLP-1-mediated targeting may effectively bypass the delivery of MK-801 to vagal afferent target neurons in the nucleus of the solitary tract. Accordingly, this mechanism would circumvent the blocking of distinct NMDA receptors in the brainstem that might otherwise increase food intake in response to MK-801. Supporting this hypothesis, we demonstrate that GLP-1 receptor-mediated targeting of MK-801 effectively avoids other adverse effects associated with MK-801 monotherapy, including hyperthermia and hyperlocomotion.

Multimodal weight loss compounds that integrate agonism at two or more receptor systems are emerging^27,43–45^. Here we report a unimolecular compound composed of a small-molecule antagonist and a peptide agonist for obesity treatment. Whereas previous research has demonstrated the feasibility of using peptides to target nuclear hormone receptors^46,47^, to our knowledge, this is the first single molecule that uses a G-protein-coupled-receptor targeting approach to deliver a small-molecule modulator of an ionotropic receptor. We provide evidence that this targeting concept can be used to antagonize NMDA receptors specifically in GLP-1-receptor-positive neurons to reverse obesity and correct symptoms of cardiometabolic diseases in preclinical obesity models. While the clinical evaluation of GLP-1–MK-801 for weight loss awaits, efforts to broaden the scope of developing peptide-mediated targeting of ion channels are encouraged and should not be restricted to metabolic diseases.

## Methods

### Peptide synthesis

All peptides were prepared using ChemMatrix Rink amide resin and Fmoc-based automated peptide synthesis on a Prelude X peptide synthesizer (Gyros Protein Technologies) with induction heating and oscillation mixing. All of the solutions were freshly prepared immediately before synthesis as stock solutions in DMF: Fmoc-protected amino acid (0.2 M), HCTU (0.5 M), DIPEA (1.0 M) and piperidine (20% v/v). Peptide elongation was achieved through consecutive cycles of Fmoc deprotection and coupling reactions. Fmoc deprotection was achieved with 20% piperidine in DMF (twice for 2 min, room temperature, 300 rpm shaking) and peptide couplings were performed as double or triple couplings (twice for 5 min, 75 °C, 300 rpm shaking, except for Arg and His, for which twice for 5 min, 50 °C, 300 rpm shaking) consisting of AA/HCTU/DIPEA (ratio, 1:1.25:2.5) in fivefold excess compared to the resin. Extensive DMF washes were performed after each deprotection or coupling reaction.

### Peptide cleavage and purification

Dried peptide-containing resin was suspended in 1.5 ml cleavage cocktail (2.5% DODT, 2.5% H_2_O, 2.5% TIPS in TFA) per 100 mg resin and agitated for 2 h. The peptide-containing solution was collected by filtration, reduced under a stream of nitrogen and precipitated with ice-cold ether. The crude peptide pellet was isolated by centrifugation at 3,600*g* for 10 min at 4 °C, redissolved in MeCN:H_2_O (1:1) and lyophilized. Ultraperformance LC (UPLC) and electrospray ionization LC–MS (ESI-LC–MS) analysis were conducted for the crude peptide. Purifications were conducted by preparative RP-HPLC, eluting with a linear gradient (20 ml min^−1^) and using a binary solvent system consisting of H_2_O:MeCN:TFA (buffer A, 95:5:0.1; buffer B: 5:95:0.1). The collected fractions were analysed by UPLC and ESI-LC–MS. Fractions with a purity of greater than 95% were pooled and lyophilized. All peptides and peptide drug conjugates were desalted by three consecutive cycles of redissolving the peptide in 0.001 M aqueous HCl followed by lyophilization. All peptides and conjugates used for in vitro and in vivo experiments were of >95% purity.

### Synthesis of disulfide linker functionalized (+)-MK-801 and inactive MK-801

2-Mercaptoethanol (Sigma-Aldrich) was treated with 2,2-dipyridyl disulfide (3 equivalents) (Sigma-Aldrich) in dry methanol for 2 h. After completion as monitored by UPLC–MS, the reaction was concentrated in vacuo. Purification by silica gel flash chromatography (EtOAc:CH_2_Cl_2_, 2:8) afforded intermediate 2-(pyridine-2-yldisulfaneyl)ethan-1-ol (> 95%). The compound was dissolved in dry CH_2_Cl_2_ under an N_2_ atmosphere and reacted with 4-nitrophenyl chloroformate (1.2 equivalents) for 4 h. The reaction was worked up by extraction, washing with water (×3) and brine. The organic layer was dried over anhydrous MgSO_4_, filtered and concentrated in vacuo. The crude residue was purified by silica gel flash column chromatography (*n*-heptane:EtOAc, 2:1) yielding 4-nitrophenyl (2-(pyridin-2-yldisulfaneyl)ethyl) carbonate (89%). The intermediate was finally reacted with the appropriate amine-containing drug (+)-MK-801 (2,2-diphenyl-1-amine; 1.5 equivalent) in dry DMF with addition of dry Et_3_N (3.0 equivalents) under an N_2_ atmosphere for 18 h. Purification by preparative HPLC afforded the disulfide linker functionalized (+)-MK-801 (70%) and inactive MK-801 (70%). Conjugation to the corresponding peptides was conducted using a general disulfide conjugation protocol.

### Disulfide conjugation protocol

The pure thiol-containing peptide and thiopyridyl-activated linker-functionalized MK-801 were dissolved in DMF (2 ml) and buffer consisting of 6 M guanidine and 1.5 M imidazole in H_2_O at pH 8 (200 µl) and agitated for at least 2 h. After completion, as monitored by UPLC and ESI-LC–MS, the reaction mixture was diluted with buffer A, filtered and purified directly by preparative RP-HPLC, eluting with a linear gradient (see the ‘Peptide cleavage and purification’ section).

### In vitro human plasma stability

Human plasma was preheated at 37 °C for 15 min before being spiked with a final concentration of 0.25 mM of the peptide or conjugate. The samples were collected at timepoints [0, 1, 2, 4, 8, 24 h], [0, 2, 4, 8, 24, 48 h] or [0, 4, 8, 24, 48, 72 h] depending on stability. The samples were processed by pretreatment with 6 M urea for 30 min at 0 °C followed by 20% trichloroacetic acid in acetone (wt./v%) at −20 °C overnight. The samples were centrifuged (13,400 rpm) for 30 min, and the supernatant was collected and filtered (0.2 µm syringe filter) into an LC–MS vial. The samples were analysed by reversed-phase UPLC (RP-UPLC) at 214 nm and LC–MS. The area under the curve was determined and normalized to the first timepoint. Regression lines were computed with GraphPad Prism 9.0 using the one-phase decay equation.

### In vitro stability assay with high glutathione concentration

GLP-1–MK-801 was dissolved in dimethyl sulfoxide at a concentration of 0.25 mM and diluted with a solution of 200 mM glutathione in PBS, pH 7, such that the final concentration of GLP-1–MK-801 in the assay was 100 µM. The solution was incubated at 37 °C and samples collected at timepoints [0, 1, 2, 4, 8, 12 h]. The samples were analysed using RP-UPLC at 214 nm and LC–MS. The data were normalized to the first timepoint. Regression lines were computed with GraphPad Prism 9.0 using the one-phase decay equation.

### GLP-1 receptor activation

GLP-1 receptor activation was determined using an in vitro bioluminescence resonance energy transfer (BRET)-based assay that measures changes in intracellular cyclic-AMP levels. HEK293 cells were transiently transfected with GLP-1 receptor and cyclic-AMP sensor using YFP-Epac-RLuc (CAMYEL). Cells were cultured at 37 °C in Dulbecco’s modified Eagle’s medium (DMEM) + GlutaMax 1965 (Gibco) supplemented with 10% fetal bovine serum and 1% penicillin–streptomycin in a humidified 10% CO_2_ incubator.

Cells were seeded in 96-well plates at a density of 35,000 cells per well and transiently transfected using Lipofectamine 2000 according to the manufacturer’s protocol. On the day of the assay, plates were removed from the incubator and each well was washed twice with 100 μl HBSS (Gibco, Life Technologies) and pre-incubated for 30 min at 37 °C with 85 μl HBSS per well. Luciferase substrate coelenterazine (5 μM, Thermo Fisher Scientific, C6780) was added and a baseline was measured after a 5 min incubation. The ligand mixture was added and measurements were recorded every minute for 30 min on a CLARIOstar Plus plate reader (BMG labtech). Dose–response curves were generated at equilibrium (10 min) and EC_50_ values were calculated from this.

### SPR biosensing of human serum albumin binding affinity

Interaction of peptide–drug conjugates with surface immobilized human serum albumin (HSA) (Sigma-Aldrich) was determined using surface plasmon resonance (SPR). The samples were acquired at 25 °C according to literature procedure using a Biacore X100 instrument equipped with a CM5 sensor chip (GE Healthcare Biosciences)^48^.

The system was equilibrated with 10 mM PBS at a flow rate of 20 µl min^−1^ to achieve a stable baseline. HSA was immobilized to the surface by pre-activation (1.0 EDC, 1.0 M NHS in PBS, pH 7.4, 7 min) followed by injection of HSA to flow cell 1 (30 µg ml^−1^ in 10 mM NaOAc buffer, pH 5.0, twice for 7 min). Unreacted sites were capped with ethanolamine (1.0 M ethanolamine in 10 mM PBS, pH 8.1, 7 min). Residual unreacted HSA was removed by three consecutive pulses of 9 s with 25 mM NaOH (3 µl), resulting in a surface of 14,360 RFU.

Interactions between immobilized HSA and the experimental compounds were determined using a running buffer of 10 mM PBS, 3% dimethyl sulfoxide (DMSO), pH 7.4 and a flow rate of 30 µl min^−1^. Peptides were dissolved in running buffer and the stock concentrations determined using the NanoDrop 2000 spectrophotometer (Thermo Fischer Scientific) at a wavelength of 280 nm. The samples were measured as triplicates by the same researcher, going from low to high concentrations. Compounds were injected for 150 s, and the corresponding dissociation constants were measured for 300 s. The surface was washed between samples with 25 mM NaOH for 4 s followed by equilibration with running buffer. Blanks were acquired after each triplicate measure.

### Mouse studies

All in vivo experiments were conducted according to international principles of animal care and under the approval of the Danish Ethical Committee for Animal Research and the Danish Animal Experimentation Inspectorate. Experiments were conducted using DIO male C57BL/6J mice (Janvier Labs) kept on a HFHS diet (58 kcal% fat, D12331i, Research Diets) from 8 weeks of age. Mice were maintained on the HFHS diet for a minimum of 16 weeks and had an average body weight of >45 g, before initiation of pharmacological studies. The mice were either single-housed or doubled-housed and maintained on a 12 h–12 h dark–light cycle (06:00–18:00) at 21–23 °C. Mice received once-daily sham injections with isotonic saline from 3 days before study start and were randomized to treatments on the basis of body weight at the day of study start. All compounds were administered as once-daily s.c. injections (between 15:00 and 18:00) with concomitant measurements of body weight and food intake. Vehicle was isotonic saline, which was also used for dissolving the compounds. Compounds were administered at the indicated doses at a volume of 5 µl g^−1^. For studies with a calorie-restricted and body-weight-loss-matched control group, HFHS diet (58 kcal% fat, D12331i, Research Diets) was weighed and provided as one separate pellet per mouse at the time of injection. The pellets were placed in each side of the cage for double-housed mice and the mice were treated with isotonic saline.

*db/db* mice (The Jackson Laboratory, 000697) were kept on a chow diet (Brogaarden, Altromin, 1310) and experiments were conducted on 9-week-old male mice. Grouping was based on blood glucose level (using a minimum of 11.1 mM) and body weight. Male *Mc4r-*KO mice were kept on a HFHS diet for 9 weeks and the mice were 17 weeks old at study start (The Jackson Laboratory, 032518). For electrophysiological studies, male pathogen-free mice at an age of 6–18 weeks were used for all experiments. All of the mice were housed under standard laboratory conditions (12 h–12 h on–off; lights on at 07:00) and a temperature-controlled environment with food and water available ad libitum. All experiments were performed in accordance with the guidelines established by the National Institute of Health Guide for the Care and Use of Laboratory Animals and approved by the University of Texas Institutional Animal Care and Use Committee. To identify POMC neurons and GLP-1 receptor-positive neurons, POMC humanized *Renilla* green fluorescent protein (hrGFP) (The Jackson Laboratory, 006421) and GLP-1 receptor *cre::tdTomato* (The Jackson Laboratory, 029283) mice were used. For calcium imaging studies, male Naval Medical Research Institute (NMRI) mice (Taconic Biosciences, BomTac:NMRI) were used (aged 18–28 days). Mice were bred and housed in the animal facility at the Department of Drug Design and Pharmacology, University of Copenhagen and studies were conducted under the approval of the Danish Ethical Committee for Animal Research and the Danish Animal Experimentation Inspectorate. The mice were housed in ventilated cages in a humidity- and temperature-controlled room (temperature, 22 ± 2 °C; humidity, 36–58%) with a 12 h–12 h light–dark cycle (lights on at 07:00) with ad libitum access to chow diet and tap water.

### Rat studies

Double-housed male Sprague-Dawley rats (Janvier Labs, RjHan:SD) were kept on a HFHS diet for 4 weeks from 8–10 weeks of age and had an average body weight of >500 g before initiation of pharmacological studies. The rats were housed in ventilated cages in a humidity- and temperature-controlled room (temperature, 22 ± 2 °C; humidity, 36–58%) under a 12 h–12 h light–dark cycle (lights on at 07:00) with ad libitum access to chow diet and tap water. The rats were administered once-daily s.c. injections (between 15:00 and 17:00) of 100 nmol kg^−1^ MK-801, 10 nmol kg^−1^ semaglutide, 100 nmol kg^−1^ GLP-1–MK-801 or vehicle, with concomitant measurements of body weight and food intake. Vehicle was isotonic saline and compounds were administered at a volume of 1 µl g^−1^. For the kaolin-intake study in Sprague-Dawley rats, 8-week-old double-housed male rats were used. 

For CTA and kaolin-intake studies, male Wistar rats (Janvier Labs, WistarRjHan:WI) were used. The rats were 8 weeks old at the initiation of the studies with a body weight of 250–325 g. The rats were group-housed (3–4 rats per cage) in ventilated cages in a humidity- and temperature-controlled room (temperature, 21 ± 2 °C; humidity, 50 ± 10%) under a 12 h–12 h light–dark cycle (light, 01:00 to 13:00) with ad libitum access to chow diet (Brogaarden, Altromin, 1324) and tap water. The rats were dosed by s.c. injections of 100 nmol kg^−1^ MK-801, 100 nmol kg^−1^ GLP-1, 10 nmol kg^−1^ semaglutide, 100 nmol kg^−1^ GLP-1–MK-801 or vehicle immediately before onset of the dark cycle at a volume of 2 ml kg^−1^.

### Tissue collection and processing

Mice or rats were euthanized by decapitation for collection of trunk blood and tissues. Blood was collected in EDTA-coated microvette tubes, chilled on ice and centrifuged at 3,000*g* and 4 °C for 10 min. The plasma was aliquoted and stored at −80 °C until analysis. Tissues were collected by dissection, frozen on dry-ice and stored at −80 °C until further processing.

### Body composition measurements

Body composition measurements were performed using quantitative nuclear magnetic resonance imaging (EchoMRI).

### Glucose tolerance, compound tolerance and insulin tolerance tests

Mice were fasted for 5 h before being challenged with an intraperitoneal injection of 1.75 g kg^−1^ of glucose dissolved in isotonic saline. Tail vein blood glucose concentrations were measured using a handheld glucometer (Contour XT, Bayers) at 0, 15, 30, 60 and 120 min after injection (for the *Mc4r* KO study a 90 min timepoint was also included). Compound tolerance was assessed by s.c. injection of the experimental compound followed by measurements of tail vein blood glucose concentrations using a handheld glucometer (Contour XT, Bayers) at various timepoints over the following 24 hours. For studies with measurements of plasma insulin and glucagon concentrations, plasma was collected by tail vein bleeding at timepoints 0, 60, 120 and 240 min. For insulin tolerance testing, the mice were fasted for 5 h before being challenged with an intraperitoneal injection of 0.75 U kg^−1^ of human insulin (Actrapid). Tail vein blood glucose concentrations were measured using a handheld glucometer (Contour XT, Bayers) at 0, 15, 30, 60 and 120 min after injection. For glucose-stimulated insulin secretion testing, the mice were fasted for 4 h before being challenged with intraperitoneal injections of 1.75 g kg^−1^ of glucose dissolved in isotonic saline. Tail vein blood glucose concentrations were measured using a handheld glucometer (Contour XT, Bayers) at 0, 15, 30, 60 and 120 min after injection. Plasma was collected at timepoints 0, 15 and 60 min by tail vein blood sampling and insulin concentrations were measured by enzyme-linked immunosorbent assay (ELISA; Crystal Chem Ultra Sensitive Mouse Insulin Eisa Kit, 90080).

### Rectal body temperature

Conscious mice were restrained and a high-precision thermometer (BIO-TK8851, Bioseblab) was carefully inserted half-way into the rectum. Temperature measurements of each mouse were performed by the same researcher on day 7 at timepoints 0, 20, 45 and 90 min in response to s.c. injection with 200 nmol kg^−1^ MK-801, 600 nmol kg^−1^ MK-801 or vehicle, and for measurement of rectal temperature on day 14 of mice receiving 14 days of once-daily s.c. dosing with 100 nmol kg^−1^ MK-801, 100 nmol kg^−1^ GLP-1, 100 nmol kg^−1^ GLP-1–MK-801 or vehicle.

### Plasma parameters

Plasma was sampled from a non-fasted state 2 h after the final compound administration. Plasma insulin levels were quantified using the Crystal Chem Ultra Sensitive Mouse Insulin ELISA kit (Crystal Chem Ultra Sensitive Mouse Insulin Eisa Kit, 90080). Plasma glucagon levels were quantified using the Mercodia Glucagon ELISA kit (10-1281-01, Mercodia). Plasma total cholesterol (Thermo Fisher Scientific, Infinity Total Cholesterol Reagent, TR13421), triglycerides (total glycerol and triglycerides) (Thermo Fisher Scientific, Infinity Total Triglycerides Reagent, TT22421), non-esterified fatty acids (NEFA) (Invitrogen, non-esterified free fatty acids (NEFA/FFA) Colorimetric Assay Kit, Thermo Fisher Scientific, EEA017), AST (Thermo Fisher Scientific, EEA003) and ALT (Thermo Fisher Scientific, EEA001) were quantified using enzymatic kits according to the manufacturer’s protocols.

### In vivo pharmacokinetic measurements

The pharmacokinetic assessment was conducted using a total of eight male DIO C57BL/6J mice (*n* = 4 per subgroup). Mice were administered single s.c. injections with a dose of 100 nmol kg^−1^ of the experimental compounds. Each subgroup was bled at the following timepoints: subgroup A at 15 min, 45 min, 2 h and 8 h; subgroup B at 30 min, 1 h, 4 h and 24 h.

Mouse plasma containing test substance was crashed in 96-well non-binding plates using liquid–liquid extraction with ethanol containing internal standard. The samples were then centrifuged, and the supernatants were transferred to new wells and diluted with water. The prepared mouse plasma was analysed for the test substance using LC–MS. The system consisted of a TSQ Quantis Triple Quad mass spectrometer (Thermo Fisher Scientific) equipped with a Vanquish Horizon UPLC (Thermo Fisher Scientific). RP-UPLC separation was performed on the Acquity UPLC system (Waters, column: BEH C18 1.7 µm, 2.1 × 50 mm). Mobile phase A was composed of 0.1% formic acid in water and mobile phase B was composed of 0.1% formic acid in acetonitrile. The UPLC flow rate was set to 0.3 ml min^−1^ at 60 °C using a gradient elution from 10 to 65% B over the course of 4.0 min. The gradient was then ramped from 65% B to 99% B for 0.1 min and held at 99% for 0.9 min. The mass spectrometer was operated in positive-ionization SRM mode.

### Intracerebroventricular administration study

The intracerebroventricular study was performed in HFHS-fed C57BL/6J mice that were single-housed in open cages. Mice were pretreated with lidocaine (Accord Healthcare) at the site of incision, anaesthetized with isoflurane and fixed in a stereotaxic instrument. The skin of the head was incised, a hole was drilled into the skull and a guide cannula was subsequently implanted into the lateral ventricle (26GA; PlasticOne; C2315GS-4/SPC) using stereotaxic coordinates (−0.3 mm posterior to bregma; ±1.0 mm lateral to bregma) (David Kopf Instruments). The guide cannula was held in place using UV-cured cement (G-bond and G-aenial Universal Flo, GC), and a dummy cannula (PlasticsOne, C315DCS-4/Spc, 2.5 mm) was inserted into the guide cannula to keep the cannulation closed until and in between compound infusions. Post-operatively, mice were administered s.c. injections of carprofen (5 mg kg^−1^, Pfizer) for 3 days and allowed to recover for a minimum of 7 days with daily monitoring of food intake and body weight.

After full recovery, correct cannulation was tested by observing water drinking responsiveness after infusion of 1 µl human angiotensin II (Sigma-Aldrich, A9525) at a concentration of 24 µM in artificial cerebrospinal fluid (distilled water with 125 mM NaCl, 2.5 mM KCl, 2.6 mM NaHCO_3_, 1.25 mM NaH_2_PO_4_·2H_2_O, 25 mM d-glucose monohydrate, 1 mM MgCl_2_, 2 mM CaCl_2_). Mice that did not drink within 15 min of the infusion were excluded from the study. In the afternoon on the experimental day, semaglutide (Novo Nordisk) and GLP-1–MK-801 were dissolved in isotonic saline at a concentration of 0.11 nmol µl^−1^. Before infusion, mice were relocated to new cages with fresh bedding to ensure that no remnant food pellets remained in the cage. Mice were placed onto a cage hopper and infused with semaglutide, GLP-1–MK-801 or vehicle (isotonic saline) over 60 s at a total infusion volume of 2 µl through a Hamilton syringe mounted onto an automated syringe pump (Harvard Apparatus) and a 33-gauge internal cannula (PlasticsOne, C315IS-4/SPC, 2.5 mm). The injector was kept in the guide cannula for 30–60 s after infusion stop to ensure complete infusions and to avoid backflow. Body weight and food intake were subsequently monitored daily in the afternoon. At day 6 after the first infusion, the semaglutide-treated and GLP-1–MK-801-treated mice were crossed-over in terms of treatment and administered with another single infusion of either GLP-1–MK-801 (0.22 nmol, 2 µl) and semaglutide (0.22 nmol, 2 µl), respectively, while vehicle-treated mice received vehicle treatment once again. Again, body weight and food intake were monitored once daily in the afternoon.

### Metabolic phenotyping and indirect calorimetry

Single-housed male DIO C57BL/6J mice were acclimatized to metabolic cages (16-channel Promethion, Sable Systems International) for 1 week before the start of the study. Oxygen consumption (VO_2_), carbon dioxide production (VCO_2_), RER, energy expenditure (kcal h^−1^) and locomotor activity (cm s^−1^) were recorded and collected in 15 min bins. Water and HFHS food were available ad libitum throughout the study period. New food was provided every second day. For study 1 (weight loss study of GLP-1–MK-801 relative to monotherapies), mice were randomly divided into four experimental groups (*n* = 8 mice per group) with similar mean body weights and assigned to receive once-daily s.c. injections of 100 nmol kg^−1^ MK-801, 100 nmol kg^−1^ GLP-1, 100 nmol kg^−1^ GLP-1–MK-801 or vehicle for 14 days. For study 2, mice were randomly divided into three experimental groups (*n* = 10 mice per group) such that each group had a similar mean body weight and was assigned to receive once-daily s.c. injections with 100 nmol kg^−1^ GLP-1–MK-801 or vehicle, or calorie restriction to match the weight loss trajectory of the GLP-1–MK-801 group for 10 days. In both studies, body weights and food intake were measured manually each day at the time of injection and dosing was performed at a volume of 5 µl g^−^^1^. Mice assigned to the different experimental groups were randomly distributed across two systems. Raw data for each individual mouse were analysed using the online tool CalR (CalR, v.1.3; www.calrapp.org) and visualized using GraphPad Prism. ANCOVA analyses for statistical comparison of regression lines in body weight versus average energy expenditure plots were computed using CalR.

### Open-field test

Locomotor activity was evaluated using an open-field test. Mice were acclimatized in the procedure room for 7 days before the experiments. The experiments were conducted by placing the mice in one of four 50 × 50 × 50 cm arenas immediately after compound administration, allowing their locomotion to be monitored by a ceiling-mounted Logitech C920 Pro camera (1,080 × 1,080 px, 30 fps, Logitech software). DIO mice were divided into four groups such that each group had the same average mean body weight (*n* = 8 mice), and their movements were recorded for a period of 20 min. Each run was conducted with one mouse from each treatment group and with run-to-run alternation so that treatments were equally distributed across all four arenas. Movement traces and quantification of locomotor activity (velocity and distance travelled) were obtained using Noldus EthoVision XT software (Noldus).

### CTA assay

A week before the study start (day −7), rats were single-housed with ad libitum access to chow diet and one bottle of water (food and water were placed in the cage lid so that there was room for two water bottles). The position of the water bottle was alternated daily between sides to avoid the development of a side preference. The animals were weighed and handled daily from day −3. On day −3, the rats were exposed to a bottle containing 0.1% saccharin-flavoured water with high palatability followed by s.c. administration of the experimental compounds (MK-801, GLP-1, GLP-1–MK-801 (all at doses of 100 nmol kg^−1^), semaglutide (10 nmol kg^−1^) or isotonic saline as vehicle). Then, 3 days after the first dosing, the rats were exposed to a two-bottle taste preference test, that is, a voluntary choice between tap-water or the 0.1% saccharin solution. Saccharin and water intake was monitored for 24 h followed by preference determination. These experiments were conducted at Gubra.

### Kaolin intake

For the Wistar pica study, rats were group-housed (3–4 rats per cage) and allowed to acclimatize to the HM-2 system (MBRose) for 7 days before the study start. At the initiation of the experiment, the rats were randomized such that each group had the same average body weight and was then assigned to receive once-daily s.c. injections of 100 nmol kg^−1^ MK-801, 100 nmol kg^−1^ GLP-1, 100 nmol kg^−1^ GLP-1–MK-801 or vehicle (isotonic saline). Food intake data were collected on a continuous basis from 24 h before dosing (allowing for replenishment of food as well as dosing time) and throughout the study. After the study start, food pellets were placed in one food channel of the HM-2 system and kaolin pellets in the other channel. The position of the food and kaolin was alternated every day to correct for side preference. These experiments were conducted at Gubra. For the Sprague Dawley study, rats were single housed and had ad libitum access to a chow diet, kaolin pellets (K50001, Research Diets) and tap water. The rats were habituated to kaolin for 5 days before pharmacological testing. At the initiation of the experiment, the rats were randomized such that each group had the same average body weight and was then assigned to receive once-daily s.c. injections of 100 nmol kg^−1^ MK-801, 100 nmol kg^−1^ GLP-1, 100 nmol kg^−1^ GLP-1–MK-801 or vehicle (isotonic saline) for 3 days. Body weight, chow intake and kaolin intake were measured daily, and the cage was carefully assessed for any remnant leftover food and kaolin.

### Voluntary running

Experiments were conducted using 8-week-old male C57BL/6J mice (Janvier Labs) kept on a chow diet. Mice were single-housed in cages equipped with a running wheel (23 cm in diameter, Techniplast). The amount of bedding material was reduced to avoid blockade of the running wheels. Running distance was monitored using a Sigma Pure 1 Topline 2016 computer (Sigma Sports) and, after 1 week of habituation, daily running distance was monitored for 3 days. The effect of experimental compounds on voluntary wheel running was monitored by randomizing mice to receive once-daily s.c. injections of 100 nmol kg^−1^ GLP-1–MK-801, 10 nmol kg^−1^ semaglutide or vehicle (isotonic saline) based on baseline running wheel distance measurements, such that each group had similar average running wheel distances at the day of study initiation. Running distance, food intake and body weight were measured daily before dosing of experimental compounds.

### Heart rate and blood pressure assessment

Blood pressure and electrocardiogram (ECG) were recorded in anaesthetized, lean mice. Chow-fed male C57BL/6J mice at 18 weeks of age were randomly assigned to receive once-daily s.c. injections of 100 nmol kg^−1^ MK-801 (*n* = 10), 100 nmol kg^−1^ GLP-1 (*n* = 10), 100 nmol kg^−1^ GLP-1–MK-801 (*n* = 10) or vehicle (isotonic saline, *n* = 10) for 14 days. Body weight and food intake were measured manually each day at the time of injections. The morning after the final injections, mice were anaesthetized using 2% isoflurane in a mix of O_2_ and N_2_ (30:70). The mice received an intraperitoneal administration of isotonic saline (0.5 ml) to compensate for potential fluid loss. Body temperature was monitored and kept at 36–37 °C throughout the procedure.

For heart rate measurements, a surface ECG was recorded using custom-made needle electrodes placed on each limb. For blood pressure measurements, a 0.8F pressure catheter (SPR-1000, Millar, 8410001) connected to a bioamplifier unit was inserted in the right carotid artery. The catheter tip was placed at the level of the aortic arch. Evaluation of blood pressure pulse profile was used to confirm correct positioning of the catheter tip. A PowerLab unit (PowerLab 16/35, AD Instruments) was used to record ECG and blood pressure at a sampling rate of 4 kHz. The ECG was recorded for 5 min after the mouse was anaesthetized and then again for 10 min once the blood pressure catheter was placed.

### Electrophysiology

Brain slices were prepared from adult *Glp1r-cre::tdTomato* or *Pomc-hrGFP* male mice (aged 6–18 weeks) as previously described^49–51^. In brief, male mice were deeply anaesthetized with an intraperitoneal injection of 7% chloral hydrate and transcardially perfused with a modified ice-cold artificial cerebrospinal fluid (aCSF) (described below). The mice were then decapitated and the entire brain was removed and immediately submerged in ice-cold, carbogen-saturated (95% O_2_ and 5% CO_2_) aCSF (126 mM NaCl, 2.8 mM KCl, 1.2 mM MgCl_2_, 2.5 mM CaCl_2_, 1.25 mM NaH_2_PO_4_, 26 mM NaHCO_3_ and 5 mM glucose). Coronal sections (250 mm) were cut using the Leica VT1000S Vibratome and then incubated in oxygenated aCSF (32–34 °C) for at least 1 h before recordings. The slices were bathed in oxygenated aCSF (32–34 °C) at a flow rate of ~2 ml min^−1^. All electrophysiology recordings were performed at ambient temperature.

The pipette solution for whole-cell recordings was modified to include an intracellular dye (Alexa Fluor 350 hydrazide dye) and contained: 120 mM K-gluconate, 10 mM KCl, 10 mM HEPES, 5 mM EGTA, 1 mM CaCl_2_, 1 mM MgCl_2_ and 2 mM MgATP, and 0.03 mM Alexa Fluor 350 hydrazide dye (pH 7.3). Epifluorescence was briefly used to target fluorescent cells, at which time the light source was switched to infrared differential interference contrast imaging to obtain the whole-cell recordings (Zeiss Axioskop FS2 Plus equipped with a fixed stage and a QuantEM:512SC electron-multiplying charge-coupled device camera). Electrophysiological signals were recorded using the Axopatch 700B amplifier (Molecular Devices), low-pass filtered at 2–5 kHz, and analysed offline on a PC with pCLAMP programs (Molecular Devices). To measure NMDA-induced inward current from GLP-1-receptor-positive neurons, we used magnesium-free aCSF (126 mM NaCl, 2.8 mM KCl, 2.5 mM CaCl_2_, 1.25 mM NaH_2_PO_4_, 26 mM NaHCO_3_ and 5 mM glucose) containing 10 μM CNQX and 100 μM picrotoxin. Membrane potentials and firing rates were measured from POMC neurons in brain slices. Recording electrodes had resistances of 2.5–5 MΩ when filled with the K-gluconate internal solution. The frequency and peak amplitude of excitatory neurons were analysed using the Easy electrophysiology program (Easy Electrophysiology).

Drug working concentrations and stock preparation were as follows: GLP-1–MK-801 and MK-801 (both 50 μM, dissolved in aCSF or magnesium-free aCSF), NMDA (100 μM, dissolved in magnesium free aCSF), CNQX (10 μM, dissolved in DMSO, Alomone Labs), picrotoxin (100 μM, dissolved in DMSO). The final concentration of DMSO applied to the slices was <0.05%.

A change in membrane potential was required to be at least 2 mV in amplitude in response to drug application. Membrane potential values were not compensated to account for junction potential (−8 mV). Effects of GLP-1–MK-801 on frequency (over 0.5 Hz) and synaptic activity before and during acute GLP-1–MK-801 bath application were analysed within a recording using the Kolmogorov–Smirnov test (a nonparametric, distribution-free goodness-of-fit test for probability distributions).

### Calcium imaging

Brain slices were prepared from adult NMRI male mice (aged 18–28 days). After deep anaesthesia with isoflurane (Attane Vet, 1,000 mg g^−1^, ScanVet, Piramal Critical Care) mice were decapitated and the brain was removed and submerged in ice-cold aCSF (124 mM NaCl, 5 mM KCl, 1.2 mM Na_2_HPO_4_·2H_2_O, 2.7 mM CaCl_2_·2H_2_O, 1.2 mM MgSO_4_ (anhydrous)), 10 mM dextrose, 26 mM NaHCO_3_ was adjusted to pH 7.4 and an osmolarity of 298–302 mOsm kg^−1^ after saturation with carbogen (95% O_2_/5% CO_2_). A vibratome (Leica VT1200S, Leica Biosystems) was used to obtain 250 μm thin acute brain slices containing Arc, identified by well-known landmarks. After incubation for 15 min in a 32 °C water bath and 1 h at ambient temperature, the slices were loaded with Fura-2-AM (4 mM; Hello Bio) under carbogen exposure in a 32 °C water bath for 10 min + 1 min for each PND. The slices were rinsed and placed into a chamber embedded in the stage of an Olympus BX51WI microscope (Olympus) coupled to a 12-bit CCD fluorescent camera (SensiCam, PCO imaging). A monochromator (Polychrome V, TILL Photonics, FEI) combined with a xenon light bulb provided fluorescent illumination. Protocols for fluorescence exposure of slices were controlled by software (Live Acquisition, TillVision), and analyses were conducted using Offline Analysis (TillVision).

Regions of interest were drawn around Fura-2-AM-loaded cells under ×40 magnification as well as around one region of the field devoid of cells, which was to be used as the background. The entire field of view was exposed to excitation wavelengths of 340 nm (exposure time, 50 ms) and 380 nm (exposure time, 40 ms). Each frame pair (340 nm:380 nm was collected at an interval of 4 s for 10 min of recording in total. GLP-1 or GLP-1–MK-801 (1 µM in aCSF) was bath-applied for 25 min, with recording only being conducted during the first 10 min, at which point a maximum and stable change in fluorescence was achieved. Fifteen additional minutes of application of GLP-1 or GLP-1–MK-801 were conducted without recording to minimize exposure to fluorescent light to reduce bleaching of the fluorescent indicator. Then, a 10 min recording with excitation was started, and after establishment of a baseline consisting of 10 frame pairs, NMDA (50 µM, Tocris) + GLP-1 or GLP-1–MK-801 was bath-applied.

The fluorescence intensity within each region of interest was binned at 2 × 2 pixels and averaged. A ratio of fluorescence intensities measured at 340 nm and 380 nm was calculated minus the background fluorescence. The peak amplitude of a change in fluorescence induced by GLP-1, GLP-1–MK-801 or NMDA was calculated by taking an average of ten datapoints from the baseline (*F*_0_) and subtracting this from an average of ten data points from the peak fluorescence (Δ*F*: average amplitude_peak_ − average amplitude_baseline_), which was normalized by dividing by *F*_0_. Graphic plots were converted to a percentage defined as %Δ *F*/*F*_0_.

### RNA-seq analysis

mRNA-seq was performed by the Single-Cell Omics platform at the Novo Nordisk Foundation Center for Basic Metabolic Research. Libraries were prepared using the Universal Plus mRNA-seq protocol (Tecan) according to the manufacturer’s protocol. Libraries were quantified with NuQuant, quality checked using a TapeStation instrument (Agilent Technologies) and subjected to 52 bp paired-end sequencing on the NovaSeq 6000 system (Illumina). For differential expression testing, the R package DESeq2 (v.1.30.1) was used to identify differentially expressed genes. *P* values were adjusted for multiple testing using the Benjamini–Hochberg post hoc method. For functional enrichment analysis, the R package gprofiler2 (v.0.2.0) was used to identify enriched functional terms (GO:MF, GO:BP, GO:CC, KEGG pathways and REACTOME pathways). The gene-set enrichment analysis was performed with the parameters ‘exclude_iea’ set to true and ‘correction method’ set to Benjamini-Hochberg. SynGO enrichment analyses were conducted using the online tool https://syngoportal.org/ with the background set to brain expressed and using differentially expressed genes (*P* < 0.05). The following transcripts are not depicted in Fig. 3e: *gh*, s*carna13* and CT010467.1.

### MS-based proteomics

Hypothalamic tissue was powdered and lysed (lysis buffer 50 mM Tris, 4% SDS buffer, pH 8.5) using BeatBox homogenizer (PreOmics). Protein lysates were boiled at 95 °C for 10 min on a thermomixer (Thermo Fisher Scientific) and sonicated on the Bioruptor (Diagenode) system. Proteins were digested into peptides using a high-throughput automated version of the protein aggregation capture workflow^52^ on the KingFisher Flex Purification System (Thermo Fisher Scientific). Proteins were on-bead digested overnight in a solution containing LysC and trypsin at 37 °C. The resulting tryptic peptides were desalted using in-house-crafted SDB-RPS StageTips and 200 ng of peptides were loaded in Evotips (Evosep) according to the manufacturer’s instructions.

Desalted peptides were separated on the Pepsep (15 cm, 150 μM inner diameter) column packed with C18 beads (1.9 μm; Bruker) on the Evosep ONE HPLC system using the ‘30 samples per day’ method, then injected through a CaptiveSpray ion source and 20 μm emitter into a timsTOF Pro 2 mass spectrometer (Bruker) operated in diaPASEF mode. The resulting MS raw files were processed with the DIA-NN software v.1.876 in a library-free manner, using a *Mus Musculus* FASTA file from UniProt (December 2023). Proteotypic peptides were used for protein group quantification, under double-pass mode neural network configuration. ‘Robust LC (high accuracy)’ was chosen as the quantification strategy and the match between runs options was enabled. The rest of the parameters were set as the default, which included precursor FDR set to 1% and peptide length of 7–30 amino acids. For differential expression analysis, the R package limma (v.3.54.2) was used to identify differentially expressed proteins. *P* values were adjusted for multiple testing using the Benjamini–Hochberg post hoc method. For functional enrichment analysis, enriched gene sets were determined by applying the same workflow used for RNA, using the R package gprofiler2 (v.0.2.0). SynGO enrichment analyses were conducted using the online tool https://syngoportal.org/ with the background set to brain expressed and using differentially expressed proteins (*P* < 0.05).

### cFOS whole-brain imaging

Lean male C57BL/6J mice (aged 8 weeks) maintained on a chow diet (Brogaarden, Altrumin, 1310) were randomized 4 days before the study start and treated with once-daily s.c. mock dosing with isotonic saline. Study 1 was conducted during the light phase. All compounds were prepared as solutions in isotonic saline and dosed as s.c. injections of 10 nmol kg^−1^ semaglutide (*n* = 8 mice), 100 nmol kg^−1^ GLP-1–MK-801 (*n* = 8 mice) or vehicle (isotonic saline, *n* = 8 mice) at a volume of 5 µl g^−1^. Study 2 was conducted during the light phase. All of the compounds were prepared as solutions in isotonic saline and dosed as s.c. injections of 100 nmol kg^−1^ MK-801 (*n* = 8 mice), 100 nmol kg^−1^ GLP-1 (*n* = 8 mice), 100 nmol kg^−1^ GLP-1–MK-801 (*n* = 8 mice) or vehicle (isotonic saline, *n* = 8 mice) at a volume of 5 µl g^−1^. One outlier was removed from the vehicle, MK-801 and GLP-1–MK-801 groups due to deviation related to tissue processing. Tissue processing and quantification of cFOS was conducted as previously described^37^. These experiments were conducted at Gubra.

Brain abbreviations are as follows: NAc, nucleus accumbens; PVH, paraventricular hypothalamic nucleus; DMH, dorsomedial nucleus of the hypothalamus; ARC, arcuate nucleus of the hypothalamus; LHA, lateral hypothalamic area; CEA, central amygdala nucleus; SNc, substantia nigra, compact part; VTA, ventral tegmental area; PB, parabrachial nucleus; NTS, nucleus of the solitary tract; DMX, dorsal motor nucleus of the vagus nerve; AP, area postrema; IMD, intermediodorsal nucleus of thalamus; PG, pontine gray; DG, dentate gyrus; MS, medial septal nucleus; LS, lateral septal nucleus; SFO, subfornical organ; PH, posterior hypothalamic nucleus; SLD, sublaterodorsal nucleus; TRN, tegmental reticular nucleus; PSTN, parasubthalamic nucleus; PS, parastrial nucleus; B, Barrington’s nucleus; PVT, paraventricular nucleus of the thalamus; RR, midbrain reticular nucleus, retrorubral area; MD, mediodoral nucleus of thalamus; SUT, supratrigmental nucleus; IRN, intermediate reticular nucleus; LC, locus ceruleus; MDRNd, medullary reticular nucleus, dorsal part; GU, gustatoty areas.

### Quantification and statistical analysis

Statistical analyses were performed using GraphPad Prism 10.1.1 (GraphPad) and figures were generated using either GraphPad Prism or CorelDraw X8 (Corel). For comparison of multiple groups, one-way ANOVA or two-way repeated measures ANOVA were used. Two-way ANOVA main effects are reported and Bonferroni post hoc multiple-comparison analyses applied when relevant for interpretation. Regression plot ANCOVA analyses of indirect calorimetry data were computed using the online tool calR (www.calR.org). For comparison of two groups, unpaired two-tailed Students *t*-tests were used. Data were evaluated for distribution patterns using tests including Shapiro-Wilk and Kolmogorov–Smirnov tests and by visual inspection of the distribution residuals. Data from designated brain regions from the cFOS 3D brain imaging study were analysed using one-way ANOVA with Dunnet’s post hoc multiple-comparison test relative to the vehicle, using a negative binomial generalized linear model to control for Gaussian distribution. However, the top 20 most statistically significantly regulated brain regions in response to treatment were analysed as previously described^37^. No statistical methods were applied to predetermine the sample size for in vivo pharmacology experiments. Data represent mean ± s.e.m.

### Reporting summary

Further information on research design is available in the Nature Portfolio Reporting Summary linked to this article.

## Online content

Any methods, additional references, Nature Portfolio reporting summaries, source data, extended data, supplementary information, acknowledgements, peer review information; details of author contributions and competing interests; and statements of data and code availability are available at 10.1038/s41586-024-07419-8.

### Supplementary information

Supplementary Fig. 1

Reporting Summary

Supplementary Table 1 Sample sizes (*n*) and statistical significance values (*P* values) for all data panels in the study.

### Source data

Source Data Fig. 1

Source Data Fig. 2

Source Data Fig. 3

Source Data Fig. 4

Source Data Fig. 5

Source Data Extended Data Fig. 1

Source Data Extended Data Fig. 2

Source Data Extended Data Fig. 3

Source Data Extended Data Fig. 4

Source Data Extended Data Fig. 5

Source Data Extended Data Fig. 6

Source Data Extended Data Fig. 7

Source Data Extended Data Fig. 8

Source Data Extended Data Fig. 9

Source Data Extended Data Fig. 10

Source Data Extended Data Fig. 11

Source Data Extended Data Fig. 12

## Data Availability

All data necessary for the conclusions of the study are provided with the Article. Genetic data generated for the bulk RNA-seq analysis of GLP-1–MK-801 versus monotherapies of nuclei from the brainstem and the nucleus accumbens are available at the Gene Expression Omnibus under SuperSeries accession number GSE245728. The MS proteomics data have been deposited to the ProteomeXchange Consortium through the PRIDE partner repository under dataset identifier PXD045816. Source data are provided with this paper.

## Code availability

The scripts used to analyse the RNA-seq data are available at GitHub (https://github.com/perslab/Petersen-Nature-2024).
