# Supplementary material for: GLP-1-directed NMDA receptor antagonism for obesity treatment
Source: Nature. 2024 May 15;629(8014):1133–41. doi: 10.1038/s41586-024-07419-8 (PMC11136670; doi:10.1038/s41586-024-07419-8)
Supplement: Supplementary file 1 — Supplementary Fig. 1 [file 41586_2024_7419_MOESM1_ESM.pdf]

nature portfolio

https://doi.org/10.1038/s41586-024-07419-8

Supplementary information

GLP-1-directed NMDA receptor antagonism for obesity treatment

In the format provided by the authors and unedited

nature portfolio

https://doi.org/10.1038/s41586-024-07419-8

Supplementary information

GLP-1-directed NMDA receptor antagonism for obesity treatment

In the format provided by the authors and unedited

Chemical structures

GLP-1 analogue. C-terminal cysteine homolog.

H—H-Aib-E-G-T-F-T-S-D-V-S-S-Y-L-E-E-Q-A-A-K-E-F-I-A-W-L-V-K-G-G-P-S-S-G-A-P-P-P-S-C—NH_2

GLP-1 analogue. C-terminal homocysteine homolog.

H—H-Aib-E-G-T-F-T-S-D-V-S-S-Y-L-E-E-Q-A-A-K-E-F-I-A-W-L-V-K-G-G-P-S-S-G-A-P-P-P-S-hCys—NH_2

GLP-1 analogue. C-terminal penicillamine homolog.

H—H-Aib-E-G-T-F-T-S-D-V-S-S-Y-L-E-E-Q-A-A-K-E-F-I-A-W-L-V-K-G-G-P-S-S-G-A-P-P-P-S-Pen—NH_2

GLP-1–MK-801 (Cys-linked).

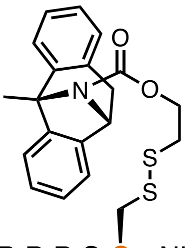

H—H-Aib-E-G-T-F-T-S-D-V-S-S-Y-L-E-E-Q-A-A-K-E-F-I-A-W-L-V-K-G-G-P-S-S-G-A-P-P-P-S-C—NH_2

GLP-1–MK-801 (hCys-linked).

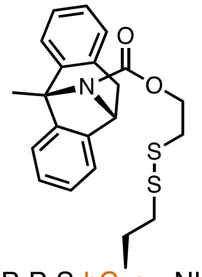

H—H-Aib-E-G-T-F-T-S-D-V-S-S-Y-L-E-E-Q-A-A-K-E-F-I-A-W-L-V-K-G-G-P-S-S-G-A-P-P-P-S-hCys—NH_2

GLP-1–MK-801 (Pen-linked).

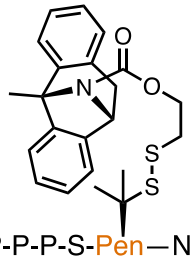

H—H-Aib-E-G-T-F-T-S-D-V-S-S-Y-L-E-E-Q-A-A-K-E-F-I-A-W-L-V-K-G-G-P-S-S-G-A-P-P-P-S-Pen—NH_2

GLP-1–inactive MK-801.

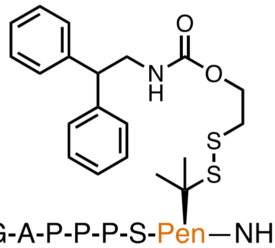

H—H-Aib-E-G-T-F-T-S-D-V-S-S-Y-L-E-E-Q-A-A-K-E-F-I-A-W-L-V-K-G-G-P-S-S-G-A-P-P-P-S-Pen—NH_2

GIP analogue.

H—Y-Aib-E-G-T-F-I-S-D-Y-S-I-A-M-D-K-I-H-Q-Q-D-F-V-N-W-L-L-A-Q-G-P-S-S-G-A-P-P-P-S-Pen—NH_2

GIP–MK-801.

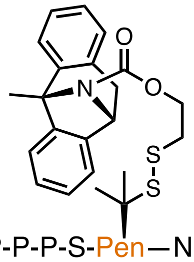

H—Y-Aib-E-G-T-F-I-S-D-Y-S-I-A-M-D-K-I-H-Q-Q-D-F-V-N-W-L-L-A-Q-G-P-S-S-G-A-P-P-P-S-Pen—NH_2

GIP/GLP-1 analogue.

H—Y-Aib-E-G-T-F-T-S-D-Y-S-I-Y-L-D-K-Q-A-A-Aib-E-F-V-N-W-L-L-A-G-G-P-S-S-G-A-P-P-P-S-Pen—NH_2

GIP/GLP-1–MK-801.

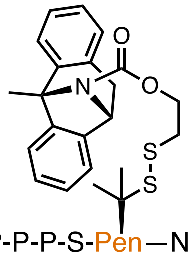

H—Y-Aib-E-G-T-F-T-S-D-Y-S-I-Y-L-D-K-Q-A-A-Aib-E-F-V-N-W-L-L-A-G-G-P-S-S-G-A-P-P-P-S-Pen—NH_2

PYY-analogue.

H—I-K-P-E-A-P-G-Pen-D-A-S-E-E-L-N-R-Y-Y-A-S-L-R-H-Y-L-N-W-V-T-R-Q-β-homoArg-Y—OH

PYY–MK-801.

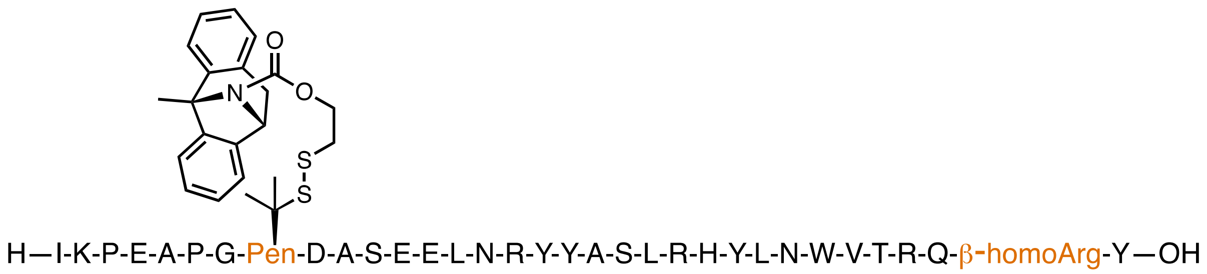

Supplementary Fig. 1 | Structures of chemical compounds. Chemical structures of GLP-1, GIP, GIP/GLP-1 and PYY analogues, and GLP-1–MK-801, GIP–MK-801, GIP/GLP-1–MK-801 and PYY–MK-801 conjugates.
