# Supplementary material for: GLP-1-directed NMDA receptor antagonism for obesity treatment
Source: Nature. 2024 May 15;629(8014):1133–41. doi: 10.1038/s41586-024-07419-8 (PMC11136670; doi:10.1038/s41586-024-07419-8)
Supplement: Supplementary file 2 — Reporting Summary [file 41586_2024_7419_MOESM2_ESM.pdf]

nature portfolio

Corresponding author(s): Christoffer Clemmensen

Last updated by author(s): May 14, 2024

Reporting Summary

Nature Portfolio wishes to improve the reproducibility of the work that we publish. This form provides structure for consistency and transparency in reporting. For further information on Nature Portfolio policies, see our Editorial Policies and the Editorial Policy Checklist.

Statistics

For all statistical analyses, confirm that the following items are present in the figure legend, table legend, main text, or Methods section.

n/a	Confirmed	
☐	☒	The exact sample size (n) for each experimental group/condition, given as a discrete number and unit of measurement
☐	☒	A statement on whether measurements were taken from distinct samples or whether the same sample was measured repeatedly
☐	☒	The statistical test(s) used AND whether they are one- or two-sided<br>*Only common tests should be described solely by name; describe more complex techniques in the Methods section.*
☒	☐	A description of all covariates tested
☐	☒	A description of any assumptions or corrections, such as tests of normality and adjustment for multiple comparisons
☐	☒	A full description of the statistical parameters including central tendency (e.g. means) or other basic estimates (e.g. regression coefficient) AND variation (e.g. standard deviation) or associated estimates of uncertainty (e.g. confidence intervals)
☐	☒	For null hypothesis testing, the test statistic (e.g. F, t, r) with confidence intervals, effect sizes, degrees of freedom and P value noted<br>*Give P values as exact values whenever suitable.*
☒	☐	For Bayesian analysis, information on the choice of priors and Markov chain Monte Carlo settings
☒	☐	For hierarchical and complex designs, identification of the appropriate level for tests and full reporting of outcomes
☒	☐	Estimates of effect sizes (e.g. Cohen's d, Pearson's r), indicating how they were calculated

Our web collection on statistics for biologists contains articles on many of the points above.

Software and code

Policy information about availability of computer code

Data collection	Noldus EthoVision XT was used for open field test recordings
Data analysis	GraphPad Prism (version 10.1.1) was used for statistical analyses<br><br>CalR online tool (version 1.3) was used for ANCOVA statistical analyses<br><br>Noldus EthoVision XT was used for open field test analyses<br><br>RNA Sequencing: Messenger RNA-sequencing was performed by the Single-Cell Omics platform at the Novo Nordisk Foundation Center for Basic Metabolic Research. Libraries were prepared using the Universal Plus mRNA-seq protocol (Tecan, US) according to manufacturer's protocol. Libraries were quantified with NuQuant, quality checked using a TapeStation instrument (Agilent Technologies, US) and subjected to 52-bp paired-end sequencing on a NovaSeq 6000 (Illumina, US). Differential expression testing: The R package DESeq2 (v. 1.30.1) was used to identify differentially expressed genes. P values were adjusted for multiple testing using the Benjamini-Hochberg post hoc method. Functional enrichment analysis: The R package gprofiler2 (v.0.2.0) was used to identify enriched functional terms (GO:MF, GO:BP, GO:CC, KEGG pathways, and REACTOME pathways). The gene-set enrichment analysis was carried out with the parameters 'exclude_iea' set to true and 'correction method' set to Benjamini-Hochberg. SynGO enrichment analysis: SynGO enrichment analyses were conducted using the online tool syngoportal.org with background set to brain expressed and using differentially expressed genes (P < 0.05).<br><br>Mass spectrometry-based proteomics: Differential expression analysis: The R package limma (v 3.54.2) was used to identify differentially expressed proteins. P values were adjusted for multiple testing using the Benjamini-Hochberg post hoc method. Functional enrichment analysis: Enriched gene sets were determined by applying the same workflow utilized for RNA, using the R package gprofiler2 (v.0.2.0). SynGO

enrichment analysis: SynGO enrichment analyses were conducted using the online tool syngoportal.org with background set to brain expressed and using differentially expressed proteins ($P < 0.05$).

SynGO portal (version 1.1) was used for SynGO data analysis

For manuscripts utilizing custom algorithms or software that are central to the research but not yet described in published literature, software must be made available to editors and reviewers. We strongly encourage code deposition in a community repository (e.g. GitHub). See the Nature Portfolio guidelines for submitting code & software for further information.

Data

Policy information about availability of data

All manuscripts must include a data availability statement. This statement should provide the following information, where applicable:

- Accession codes, unique identifiers, or web links for publicly available datasets
- A description of any restrictions on data availability
- For clinical datasets or third party data, please ensure that the statement adheres to our policy

All data necessary for the conclusions of the study are available in the main text, figures, and extended data. The scripts used to analyze the RNA-seq data can be found at https://github.com/perslab/Petersen-Nature-2024. Genetic data generated for the bulk RNA-sequencing of GLP-1/MK-801 versus monotherapies of nuclei from the brainstem and the nucleus accumbens are available in the GEO under SuperSeries accession number GSE245728. The mass spectrometry proteomics data have been deposited to the ProteomeXchange Consortium via the PRIDE partner repository with the dataset identifier PXD045816. Source data are provided with this paper.

Human research participants

Policy information about studies involving human research participants and Sex and Gender in Research.

Reporting on sex and gender	n/a
Population characteristics	n/a
Recruitment	n/a
Ethics oversight	n/a

Note that full information on the approval of the study protocol must also be provided in the manuscript.

Field-specific reporting

Please select the one below that is the best fit for your research. If you are not sure, read the appropriate sections before making your selection.

☒ Life sciences ☐ Behavioural & social sciences ☐ Ecological, evolutionary & environmental sciences

For a reference copy of the document with all sections, see nature.com/documents/nr-reporting-summary-flat.pdf

Life sciences study design

All studies must disclose on these points even when the disclosure is negative.

Sample size

No statistical methods were applied to predetermine sample size for in vivo pharmacology experiments. Sample size were determined based on previous experience with these types of experiments (PMID: 38427737, PMID: 37148870, PMID: 35995995, PMID: 30459311)

Data exclusions

Fig. 1o,p: One mouse in calorie restriction group excluded due to CO_2 sensor related deviation. Fig. 3l-r: One mouse in MK-801 group was sacrificed due to illness following the intraperitoneal glucose tolerance test on day 9.

Fig. 4g, h: One sample was lost in semaglutide group

Fig. 4k: Saccharin intake data was not obtained for one mouse in GLP-1 group due to technical error with one sensor.

Fig. 5g-i: One mouse was removed from the GLP-1/MK-801 group due to tissue processing related deviation.

Extended Data Fig. 1a-d, One mouse and cage was excluded from vehicle group due to illness (severe constipation).

Extended Data Fig. 3f, g, For MRI, two values were not registered from MK-801 group, two values were not registered from GLP-1 group and two values were not registered from GLP-1/MK-801 group due to MRI instrumental error.

Extended Data Fig. 3o-s, One mouse in MK-801 group was excluded due to sensor related deviation.

Extended Data Fig. 4d, e, One datapoint in vehicle group was outside of the assay range

Extended Data Fig. 4f, g, One datapoint in vehicle group was outside of the assay range.

Extended Data Fig. 4m, n, One datapoint in vehicle, one datapoint in MK-801 and one datapoint in GLP-1/MK-801 were outside assay range.

Extended Data Fig. 4t, Four datapoints were excluded for one cage in vehicle group due to shredding on days 11-14.

Extended Data Fig. 5b , Two samples were removed from vehicle group: One sample was non-detectable and for another sample the registered activity was a significant outlier (Grubbs test).

Extended Data Fig. 5d-j, One mouse was euthanized on day 12 in MK-801 group due to sickness.

Extended Data Fig. 9c, One food monitor did not work for one mouse in vehicle group.

Extended Data Fig. 9d, One datapoint in GLP-1/MK-801 was omitted due to extensive shredding.

Extended Data Fig. 9h, Two data points omitted from analysis in vehicle group due to extensive shredding

Extended Data Fig. 9i-m, One mouse in vehicle group was excluded from analysis due to sickness.

Extended Data Fig. 9l, One datapoint in vehicle group was excluded due to the running wheel malfunction between day -1 to day 0

Extended Data Fig. 11a-j, One mouse in groups: MK-801, GLP-1/MK-801 and vehicle excluded from analysis due to sample processing related deviation

Extended Data Fig. 11k-o, One mouse in GLP-1/MK-801 group excluded due to sample processing related deviation

Replication

Main 14-day weight loss studies with GLP-1/MK-801 (Fig. 1c) has been conducted a total of 4 times with the same magnitiude of weight loss.

The study with GLP-1/'inactive MK-801' has been conducted twice, demonstrating equipotent effect with the GLP-1 monotherapy control, while GLP-1/MK-801 delivered a significantly greater weight loss. The study was conducted with compounds blinded.

Whole brain c-Fos imaging of GLP-1/MK-801 was performed twice.

In general, animal studies have been conducted by multiple researchers, several of which blinded to the treatment groups, to ensure reproducibility.

Randomization

Before initiation of pharmacological studies, mice were randomized and separated into different treatment groups, such that each treatment group had approximately the same average mean body weight.

For the study in db/db mice, grouping was based on both basal blood glucose levels and body weight.

Blinding

For c-Fos whole-brain imaging experiments and in vitro GLP-1R signaling experiments, the investigators were blinded to the treatment groups.

The remaining studies were not blinded to the investigators but were conducted according to standardized protocols and procedures.

Reporting for specific materials, systems and methods

We require information from authors about some types of materials, experimental systems and methods used in many studies. Here, indicate whether each material, system or method listed is relevant to your study. If you are not sure if a list item applies to your research, read the appropriate section before selecting a response.

Materials & experimental systems

n/a	Involved in the study
☒	☐ Antibodies
☐	☒ Eukaryotic cell lines
☒	☐ Palaeontology and archaeology
☐	☒ Animals and other organisms
☒	☐ Clinical data
☒	☐ Dual use research of concern

Methods

n/a	Involved in the study
☒	☐ ChIP-seq
☒	☐ Flow cytometry
☒	☐ MRI-based neuroimaging

Eukaryotic cell lines

Policy information about cell lines and Sex and Gender in Research

Cell line source(s)

HEK293 cells (Sigma Aldrich, cat. no. 85120602)

Authentication	Cell lines were not authenticated
Mycoplasma contamination	All cell lines tested were negative of mycoplasma contamination
Commonly misidentified lines (See ICLAC register)	HEK293 cells were used because of their reliable growth and propensity for transfection

Animals and other research organisms

Policy information about studies involving animals; ARRIVE guidelines recommended for reporting animal research, and Sex and Gender in Research

Laboratory animals	Most experiments were conducted using diet-induced obese (DIO) male C57BL/6J mice (Janvier Labs, Le Genest-Saint-Isle, France) kept on a high-fat, high-sugar diet (HFHS) (58 kcal% fat, #D12331i, Research Diets, New Brunswick, NJ, USA) from 8 weeks of age. Mice were maintained at ad libitum HFHS diet for a minimum of 16 weeks and had an average body weight of >45 g, before initiation of pharmacological studies.<br><br>db/db mice (The Jackson Laboratory, stock no. 000697) were kept on a chow diet (Altromin #1310, Lage, Germany) and experiments were conducted on 9-week-old male mice.<br><br>Male MC4R KO mice were kept on a HFHS diet for 9 weeks and studies initiated using 12-week-old male mice (The Jackson Laboratory, stock no. 032518).<br><br>For electrophysiological studies, male pathogen-free mice at an age of 6-18 weeks were used for all experiments. To identify POMC neurons and GLP-1 receptor-positive neurons, POMC humanized Renilla green fluorescent protein (hrGFP) (The Jackson Laboratory, stock no. 006421) and GLP-1 receptor cre::tdtomato (The Jackson Laboratory, stock no. 029283) mice were utilized.<br><br>For calcium imaging studies, male Naval Medical Research Institute (NMRI) mice (Taconic Biosciences, stock no. BomTac:NMRI) were used (18 – 28 days).<br><br>Male Sprague Dawley rats (Janvier Labs, Le Genest-Saint-Isle, Frances) were kept on a HFHS diet for 4 weeks from 8-10 weeks of age and had an average body weight of >500 g.<br><br>Male Sprague Dawley rats (Janvier Labs, Le Genest-Saint-Isle, Frances) kept on chow diet and used for kaolin pica experiments from 8 weeks of age and had an average body weight of 280-350 g.<br><br>For conditioned taste aversion and kaolin intake studies, male Wistar rats (Janvier Labs, stock no. WistarRjHan:WI, France) were used. The rats were 8 weeks at initiation of studies and body weight of 250-325g.
Wild animals	The study did not involve wild animals
Reporting on sex	All animal studies were conducted using male rodents.
Field-collected samples	The study did not involve field-collected samples
Ethics oversight	Unless otherwise specified, all in vivo experiments were conducted according to international principles of animal care and under the approval of the Danish Ethical Committee for Animal Research and Danish Animal Experimentation Inspectorate (2018-15-0201-01457, 2023-15-0201-01442 and 2023-15-0201-01530).<br><br>Electrophysiological studies were performed in accordance with the guidelines established by the National Institute of Health Guide for the Care and Use of Laboratory Animals and approved by the University of Texas Institutional Animal Care and Use Committee.<br><br>Mice were bred and housed in the animal facility at the Department of Drug Design and Pharmacology, University of Copenhagen and studies conducted under the approval of the Danish Ethical Committee for Animal Research and the Danish Animal Experimentation Inspectorate.

Note that full information on the approval of the study protocol must also be provided in the manuscript.
